# Acceptability and perceptions of personalised risk-based cancer screening among health-care professionals and the general public: a systematic review and meta-analysis

**DOI:** 10.1016/S2468-2667(24)00278-0

**Published:** 2025-02

**Authors:** Naomi Q P Tan, Renu S Nargund, Elisa E Douglas, Maria A Lopez-Olivo, Paul J Resong, Sayaka Ishizawa, Sara Nofal, Kate Krause, Robert J Volk, Iakovos Toumazis

**Affiliations:** Division of Oncology, Robert Wood Johnson Medical School, Rutgers University, New Brunswick, NJ, USA (N Q P Tan PhD); Rutgers Cancer Institute, New Brunswick, NJ, USA (N Q P Tan); Department of Health Services Research (N Q P Tan, R S Nargund MSc, E E Douglas PhD, M A Lopez-Olivo MD PhD, P J Resong BS, S Ishizawa PhD, S Nofal MD PhD, Prof R J Volk PhD, I Toumazis PhD) and Research Medical Library (K Krause MS), The University of Texas MD Anderson Cancer Center, Houston, TX, USA; Reno School of Medicine, University of Nevada, Reno, NV, USA (P J Resong)

## Abstract

**Background:**

Personalised risk-based screening (PRBS) can enhance the efficiency of cancer screening programnes, but little is known about support for its implementation among the general public and health-care professionals. We aimed to summarise the acceptability and perceptions of PRBS for breast, cervical, colorectal, lung, and prostate cancer screening among these groups.

**Methods:**

We conducted a systematic review and meta-analysis of original research studies reporting on breast, cervical, colorectal, lung, and prostate cancer screening; personalised risk assessments to guide PRBS; and the acceptability of and receptibility towards these approaches among the general public, health-care professionals, or both. We searched MEDLINE, Embase, Cochrane Central, PsycINFO, and CINAHL Plus for articles published between Jan 1, 2010, and April 30, 2024. Studies not reporting on the outcomes of interest and with insufficient data for analysis were excluded. Six reviewers independently screened articles, and risk of bias was assessed using the Mixed Methods Appraisal Tool. Qualitative data were analysed thematically. Quantitative data were analysed with use of random-effects meta-analysis for outcomes that had at least two studies. The study protocol was registered at PROSPERO, CRD42022354287.

**Findings:**

Our search identified 4491 unique records. After screening, 63 studies were included in our analysis, of which 36 (57%) included the general public, 21 (33%) included health-care professionals, and six (11%) included both. The majority of studies focused on breast cancer screening (43 [68%] studies), and were from North America (28 [44%]) and Europe (28 [44%]). Qualitative findings were analysed thematically, and the extracted quantitative findings were synthesised under the following topics: acceptability and perceptions of personalised risk assessments among the general public; acceptability and perceptions of PRBS among the general public; acceptability and perceptions of PRBS among health-care professionals; and barriers and facilitators to PRBS implementation among health-care professionals. The general public and health-care professionals generally found PRBS acceptable, but they needed more information about how risk was calculated and the accuracy of risk scores. Additionally, both groups were cautious about reducing screening frequencies for individuals at low risk and cited barriers such as the time and resources needed to implement an effective PRBS programme. The pooled estimate for acceptability of PRBS was 78% (95% CI 66–88) among the general public and 86% (64–99) among health-care professionals.

**Interpretation:**

The general public and health-care professionals both viewed personalised risk assessments as providing valuable information and PRBS as a logical next step to increase the quality of patient care and improve cancer mortality. However, implementation barriers at the public, health-care professional, and system level need to be addressed.

## Introduction

In most countries, eligibility for population-based cancer screening programmes for breast, cervical, and colorectal cancers is based on age and sex.^1–4^ Prostate cancer screening is recommended on an individual basis after shared decision making.^5^ In the USA, eligibility for lung cancer screening is based on age, smoking status, and pack-year smoking history.^6^ Although population-based screening programmes can lower cancer mortality, increasing awareness of varying probabilities of cancer development at the individual level have led to discussions about the limitations of the current one-size-fits-all approach.^7^ Population-based screening programmes do not account for differing risk–benefit ratios among individuals, and can lead to underscreening of individuals at high risk, overscreening of individuals at low risk, overdiagnosis, or overtreatment.^8,9^ The lifetime risk of receiving a false-positive result for at least one of the five cancers considered in this study is estimated to be almost 40% for men and 85% for women.^10^ These insights have fuelled a shift towards personalised risk-based screening (PRBS) to enhance the value of early detection.

PRBS is defined as an approach that considers individual risk factors—such as genetics, environmental exposures, and family history—to customise screening schedules.^7^ There is emerging evidence that validated risk prediction models have sufficient accuracy when selecting individuals for screening, in some cases outperforming population-based recommendations.^11–15^ PRBS can also make screening accessible and enhance efficiency of programmes by allocating limited resources to individuals who are more likely to benefit, although there remain barriers to accessing preventive services, particularly in vulnerable groups. For example, approximately 25% of lung cancers are among never-smokers,^16^ who are not eligible for lung cancer screening per the 2021 US Preventive Services Task Force recommendations. Moreover, evidence suggests that PRBS could lead to considerable health-care savings without compromising the quality of cancer care.^17,18^

Implementing PRBS in routine practice requires buy-in of the general public and health-care professionals. However, few reviews have explored the acceptability and perceptions of PRBS among these groups,^19,20^ with most reviews focusing on specific cancers.^21,22^ The objective of this systematic review is to summarise the acceptability and perceptions of PRBS (eg, use of risk calculators, tailoring screening pathways, and PRBS implementation) among the general public and health-care professionals for five cancer sites with established screening programmes, and provide recommendations on ways to address barriers and leverage facilitators to chart a way forward for PRBS to become a part of routine clinical practice.

## Methods

### Search strategy and selection criteria

We conducted a systematic review and meta-analysis to evaluate evidence on the acceptability and perceptions of personalised risk-based cancer screening among health-care professionals and the general public. Findings are reported following the PRISMA statement.^23^ The study protocol was registered at PROSPERO (CRD42022354287) on Aug 30, 2022, and is available online.

Eligible studies for the systematic review were original research studies reporting on: (1) breast, cervical, colorectal, lung, and prostate cancer screening (prostate cancer was included because it is offered as an option after shared decision making); (2) personalised risk assessments (PRAs) to guide PRBS, including use of a risk calculator and hypothetical PRBS scenarios; and (3) the acceptability of and receptivity towards these approaches among the general public, health-care professionals, or both. Where possible, we followed the operationalisation of acceptability used in the original study. Otherwise, we followed the theoretical framework of acceptability, which defines acceptability as “the extent to which people delivering or receiving a healthcare intervention consider it to be appropriate”.^24^ For health-care professionals, we included any clinical professionals (eg, physicians and nurses) across specialties (eg, primary care, radiology, and pulmonology), and non-clinical professionals who were involved in the implementation of screening or who were included as health-care professionals in the studies (eg, practice managers and researchers). We included studies with any type of comparator and studies with no comparators. Studies not reporting on the outcomes of interest and with insufficient data for analysis were excluded.

We searched Ovid MEDLINE, Ovid Embase, Cochrane Central, Ovid PsycINFO, and CINAHL Plus for articles published from Jan 1, 2010, to April 30, 2024. The search started in 2010 because the Scopus keyword search showed that there was a major increase in published studies on PRBS starting from 2010.

An information specialist with expertise in systematic reviews (KK) built the search strategy (appendix pp 2–4), adhering to the PRISMA-S1 checklist. Searches were restricted to English language articles.

EndNote X9 (Clarivate, Philadelphia, PA, USA) was used for citation management. Deduplication was performed manually in Endnote. Covidence was used for screening, quality appraisal, and data abstraction. Six coauthors (NQPT, EED, PJR, RSN, SN, and SI) screened studies for inclusion. The title and abstract of each reference were independently screened by two reviewers according to the inclusion and exclusion criteria. Studies that passed the title and abstract review were retrieved for full-text screening by two independent reviewers. Disagreements were resolved through discussion or by a third coauthor (IT, MAL-O, or RJV).

### Data analysis

Three reviewers (NQPT, EED, and RSN) extracted data on the study and participant characteristics using a standardised pilot-tested form (appendix pp 5–6). Data extractions were completed by one coauthor (NQPT, EED, or RSN) and then checked by a second coauthor (NQPT, EED, SN, or SI) for accuracy (ie, correct information extracted and no relevant information missed). Disagreements were resolved through discussion or by a third coauthor (IT or MAL-O).

Study and participant characteristics were collected from each included study. For outcomes, we decided a priori to focus on perceptions and acceptability of PRBS among the general public and health-care professionals. Examples of acceptability include participants’ perceptions of how good an idea it is to personalise risk estimates or health-care professional interest in adopting PRBS. After familiarising ourselves with the quantitative data, we decided to also extract the following outcome categories for participants: willingness to engage in PRA, satisfaction with PRBS, intended adherence to recommended screening protocols, and behavioural modifications in response to PRAs (eg, changing screening protocols as recommended). For health-care professionals, we additionally extracted: knowledge, utilisation, and self-efficacy, and perceived barriers and facilitators to implementing PRBS. We collected all results compatible with each outcome category irrespective of the measurement type, timepoints, or analytical methods.

We used the Mixed Methods Appraisal Tool to assess risk of bias (appendix p 7).^25^ Reviewers rated each study on seven appraisal questions with response options of yes (recoded as 1), no (recoded as 0), and cannot tell (recoded as 0). Five coauthors (NQPT, EED, RSN, SN, and SI) independently assessed the risk of bias of each included study. Disagreements were resolved through discussion or by a third author (IT, MAL-O, or RJV). No studies were excluded based on the quality appraisal score.

Qualitative data (from qualitative and mixed methods studies) were analysed thematically using Dedoose version 9.2.4, a qualitative analysis software. First, one coauthor (NQPT) assigned initial codes to each relevant finding and excerpt to summarise the content. Initial codes were organised into broader descriptive codes, and then into four broader thematic topics reflecting patterns observed in the qualitative data. A second coauthor (IT) evaluated the first reviewer’s coding in between steps, checking whether any excerpts were missed; that coded excerpts were relevant to the outcomes of interest; and that the assigned codes accurately reflected the meaning behind the finding. Conflicts were resolved through discussion.

Quantitative data (from quantitative and mixed methods studies included in the review) were grouped according to the outcome categories listed earlier. Where possible, we followed the outcome categories used by the original authors to reflect their rationale for selecting that measure. We then conducted a meta-analysis for outcomes that had at least two studies, using Stata version 18 and the metaprop command. All pooled proportions were calculated using the Freeman-Tukey double arcsine transformation to stabilise variances and a meta-analysis was conducted using inverse variance weights. We used random-effects models to calculate more conservative pooled estimates. The pooled estimates and their 95% CI boundaries were then back-transformed into proportions. Study heterogeneity was assessed using the *I*^2^ statistic and subsequent χ^2^ test. Quantitative variables with insufficient data for calculating pooled estimates were summarised descriptively.

Given that broad thematic topics were already developed in the qualitative coding process, we used these topics as a starting point for comparing and synthesising the qualitative and quantitative data. Upon comparison, we found common findings in the qualitative and quantitative data. We did not find divergences that resulted in inconsistencies in interpretation of the data. Findings that were unique to either the qualitative or quantitative data could be grouped to provide a multi-faceted perspective of the theme. Hence, the qualitative and quantitative findings were synthesised under the four qualitative topics.

Finally, we conducted subgroup analysis by cancer site, geographical region, publication year, sex of participants, and age of participants (appendix p 8).

### Role of the funding source

The funders of the study had no role in study design, data collection, data analysis, data interpretation, or writing of the report.

## Results

**Figure 1:**
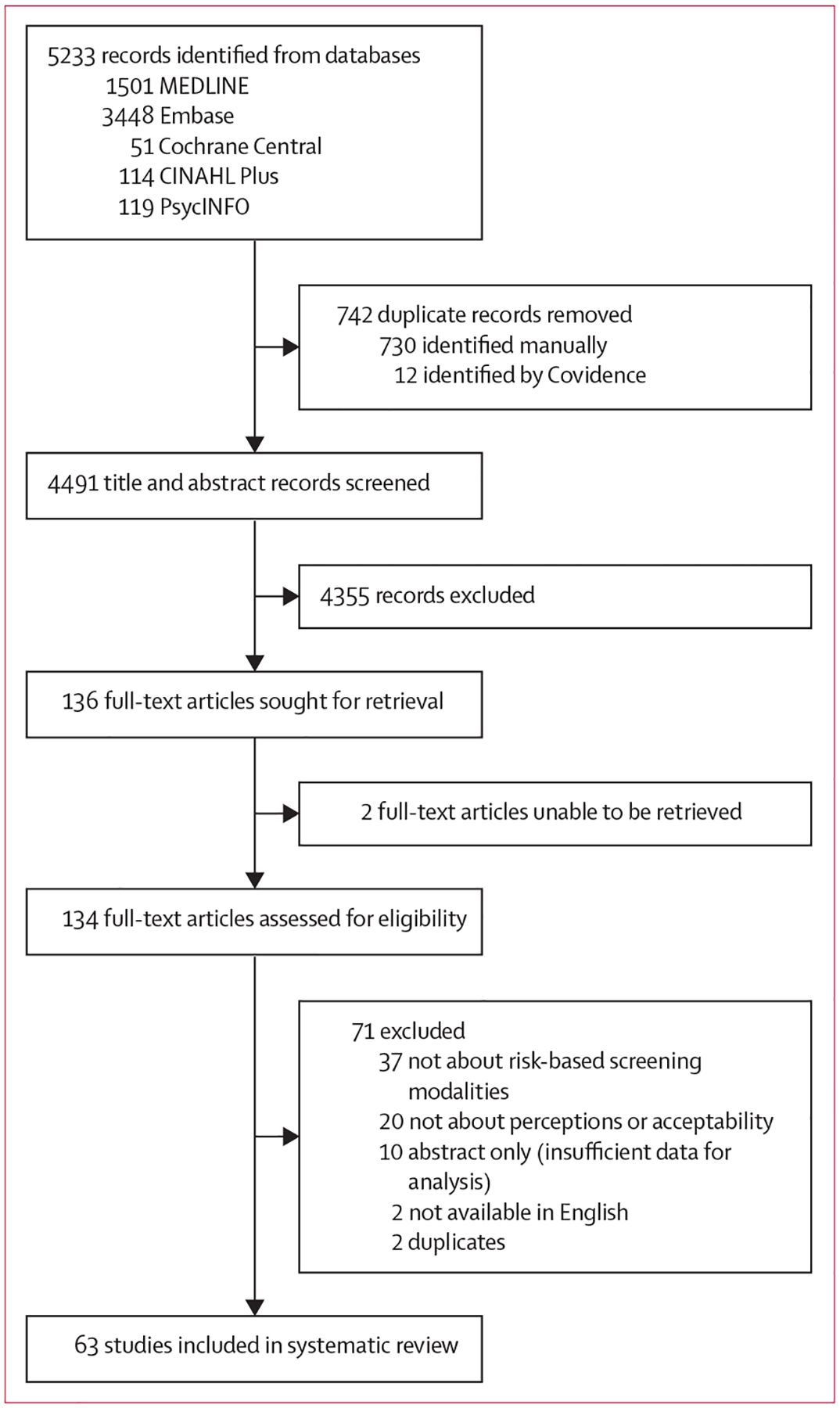
Study selection

Our search identified 4491 unique records for screening, of which 136 articles were identified for full-text screening. After full-text screening, 63 articles met our eligibility criteria (figure 1). A list of articles excluded at the full-text screening stage is provided in the appendix (pp 9–13).

The characteristics of the 63 included studies are summarised in the appendix (pp 14–24). For screening type, 43 (68%) studies focused on breast cancer, eight (13%) on colorectal cancer, six (10%) on lung cancer, three (5%) on prostate cancer, one (2%) on cervical cancer, one (2%) on both breast and colorectal cancers, and one (2%) on both breast and prostate cancers. 31 (49%) studies used quantitative methods, 22 (35%) used qualitative methods, and ten (16%) used mixed methods. For study population, 36 (57%) included the general public, 21 (33%) included health-care professionals, and six (11%) included both. There were 28 studies from North America (44%), 28 from Europe (44%), five from Australia (8%), and two from Asia Pacific (3%). Characteristics of the general public and health-care professional participants are provided in the appendix (pp 25–32).

The quality scores of included studies are presented in the appendix (pp 33–35). Overall, most studies (except one^26^) had clear research questions, and the collected data were consistent with their objectives (except one, which was an abstract^27^). None of the qualitative studies or randomised controlled trials were judged to be of poor quality. Common quality issues for the eight non-randomised studies were the inability to tell whether confounders were accounted for (50%, four studies)^28–31^ and whether participants were representative (38%, three studies).^28,31,32^ Common quality issues for the 19 cross-sectional studies were not assessing non-response bias (16% [three studies] did not^33–35^ and 53% [ten studies] had insufficient information^27,36–44^) and not having a representative sample (11% [two studies] did not^33,45^ and 47% [nine studies] had insufficient information^27,34,37,38,41–44,46^). Common quality issues for the ten mixed methods studies were insufficient information on inconsistencies in the quantitative and qualitative results (30%, three studies)^47–49^ and whether the quality criteria of each method were adhered to (20%, two studies).^48,50^

A funnel plot showed no asymmetry, indicating a low likelihood of small-study effects influencing the overall results (appendix p 36). The regression-based Egger test for small-study effects did not find evidence of publication bias (p=0·99).

**Figure 2:**
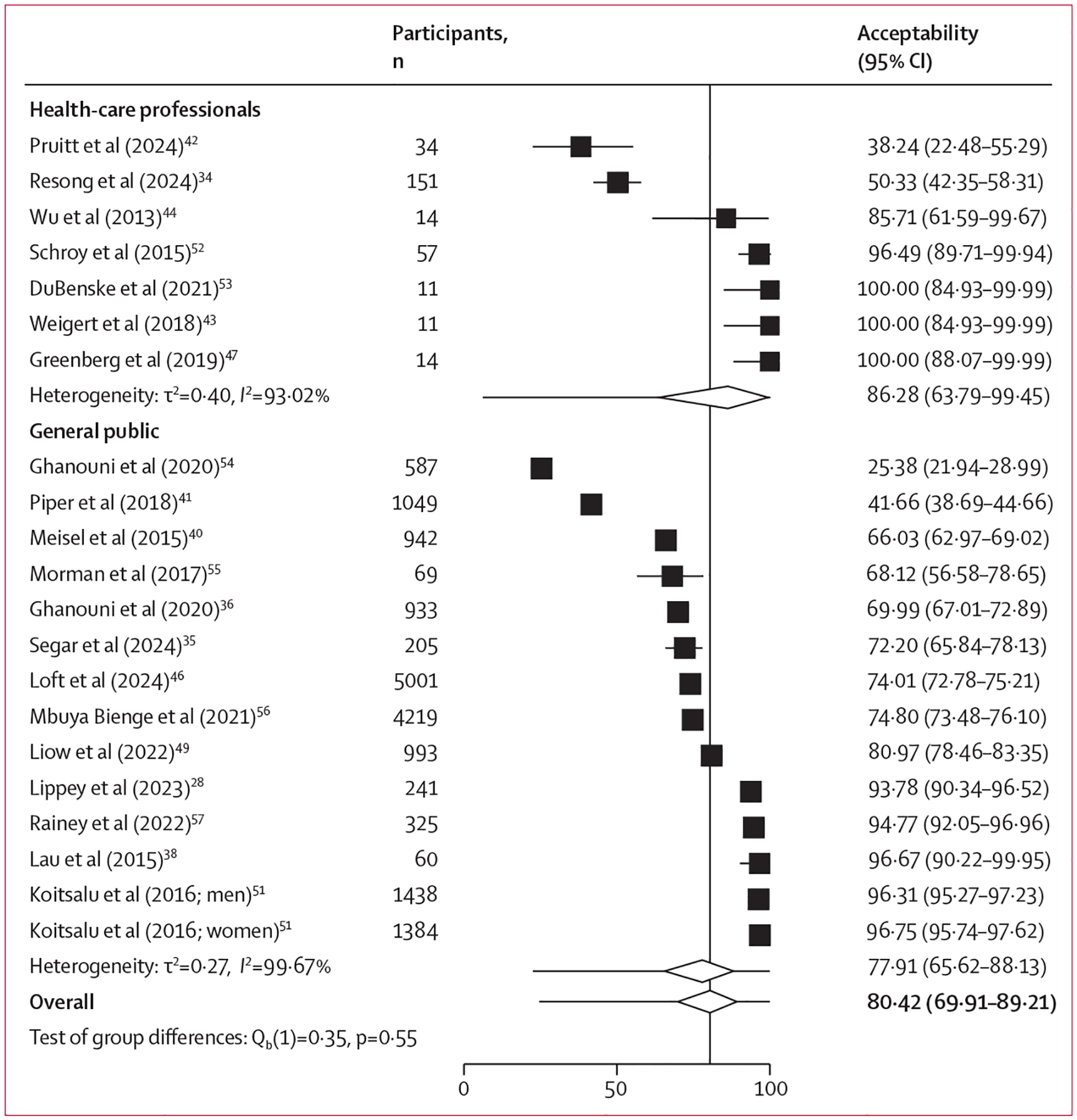
Acceptability of personalised risk-based screening among the general public and health-care professionals

Findings extracted from the qualitative and mixed method studies, as well as those from quantitative studies, are presented in the appendix (pp 37–45). Our syntheses identified four topics: acceptability and perceptions of PRAs among the general public; acceptability and perceptions of PRBS among the general public; accept ability and perceptions of PRBS among health-care professionals; and barriers and facilitators to PRBS implementation among health-care professionals. The topics and sub-themes identified from our thematic analysis of the qualitative and mixed methods studies, with supporting quotations, are presented in the appendix (pp 46–48). Pooled estimates for the quantitative outcomes are shown in the table. Forest plots for the acceptability of PRBS are shown in figure 2, and forest plots for the other outcome variables are shown in the appendix (pp 49–52).

There were 149 qualitative excerpts on the acceptability and perceptions of PRAs from 16 studies involving 730 participants from the general public.^48,49,53,58–70^ Many participants found personalised risk scores acceptable and valuable in providing important insights that could inform risk management strategies and decision making (20 excerpts, seven studies).^49,59,61,62,64,69,70^ For example, some participants reported that they might use their risk scores to inform decisions on lifestyle changes or prophylactic surgery to reduce their cancer risk. 12 excerpts (four studies) highlighted that risk scores were only useful if actionable recommendations were provided.^49,61,64,69^ Participants generally did not raise concerns about providing information to complete risk assessments (two excerpts, two studies^61,62^). Although a few participants reported that receiving a lower risk score was reassuring (five excerpts, five studies),^48,58,65,66,68^ most noted that they would be anxious waiting for the risk results and that a high-risk result would cause anxiety (15 excerpts, five studies).^49,61,66,67,69^ Others were unsure if they could trust model-estimated risk scores as risk scores can change over time (12 excerpts, 6 studies).^48,53,60,62–64^ Many participants had a high cancer risk perception given their personal and family health history. However, they reported that receiving a lower risk score would not change their risk perceptions or how often they would want to be screened (27 excerpts, six studies).^48,58,64–66,69^ Some of these participants also felt that an actual test looking for cancer was more accurate than model-estimated risk scores derived from a questionnaire.^48^

Similarly, we found that in the majority of quantitative studies with data on the acceptability and perceptions of PRAs among the general public (12 studies), a high proportion of participants were willing to engage with and perceived value in PRAs, and were comfortable providing information on or expressing a desire to know risk levels.^28,30,35,36,39,41,46,49,51,53,56,71^ Across five studies, 84% (95% CI 71–94) of participants reported being willing to engage in PRA.^30,36,46,51,71^ Across three studies, the pooled estimate for satisfaction with the PRA process was 90% (76–99).^30,31,53^ Two other studies reported high satisfaction with using the risk assessment tool,^72,73^ and one study found that participants had less decisional conflict after using a tool.^38^ 12 studies examined participants’ personal perceived risk of cancer;^27–29,31,32,39,40,46,48,55,56,58^ four found that participants consistently underestimated or overestimated their risk at baseline.^27,29,48,58^ Four studies also found that perceived risk decreased after use of a shared decision model or undergoing the PRA.^29,31,48,58^ Six quantitative studies examined how well participants comprehended and remembered the results of PRAs, reporting considerable variability.^32,39,55,57,73,74^

There were 146 qualitative excerpts on the acceptability and perceptions of PRBS from 15 studies and 702 general public participants.^49,53,59–70,75–79^ Many participants found PRBS acceptable, and cited benefits such as understanding their risk better, taking proactive action if at high risk, undergoing fewer screenings if at low risk, helping with decision making, feeling more confident in their screening decision, and providing participants who were at high risk but not eligible according to guidelines with access to screening (44 excerpts, ten studies).^49,53,59,60,62,63,66,68,69,75^ Most participants were cautious about reducing their screening frequency if their risk was low, with most being concerned that interval cancers could develop (13 excerpts, five studies).^53,60,62,63,66^ Some participants were unwilling to change their screening frequency because it brought them peace of mind (ten excerpts, three studies).^60,63,69^ Others reported that they would be willing to adopt PRBS if they were provided with compelling evidence about the accuracy of risk models (nine excerpts, three studies^59,62,63^), if participation was optional (three excerpts, two studies^59,66^), and if they were monitored by their clinician in between screenings (eg, clinical breast examinations; four excerpts, two studies^62,63^). Additionally, some participants were sceptical about risk-based screening, seeing it as motivated by cost savings (five excerpts, three studies).^60,63,66^

Overall, acceptability of PRBS among general public participants from 13 quantitative studies was 78% (95% CI 66–88; figure 2).^28,35,36,38,40,41,46,49,51,54–57^ Participants identified as being at high risk were more likely to modify their screening behaviours after receiving their score, compared with their counterparts at low risk.^28,35–38,40,46,51,56,57,71^ We observed that 86% (73–95) of participants would be willing to be screened more if categorised as high risk, whereas 57% (47–67) of participants were willing to be screened less if categorised as low risk.^28,35,36,40,46,51,56,57,71^ The pooled estimate for intended adherence to recommended screening protocols based on PRAs among participants was 31% (0–94).^54,74^

There were 114 qualitative excerpts on the acceptability and perceptions of PRBS from 16 studies and 375 health-care professionals.^26,47,50,52,53,75–85^ Overall, health-care professionals agreed that current population-based screening guidelines were insufficient and that there was a need for a more tailored approach. Benefits of PRBS reported by health-care professionals included minimising screening harms for individuals at low risk, redistributing resources, providing better care at the population level, helping individuals understand their risk, reassuring individuals or persuading them to be screened, and helping individuals with decision making (25 excerpts, nine studies).^26,50,52,75–77,79,80,85^ Additionally, health-care professionals viewed risk stratification tools as helping to facilitate conversations about screening and assisting them in communicating risk to their patients (14 excerpts, six studies).^47,53,75,78,80,85^ Despite their acceptance, health-care professionals acknowledged that it might be difficult for individuals to accept changes in screening intervals (five excerpts, three studies).^53,76,77^

Health-care professionals were cautious about implementation of PRBS. First, health-care professionals were unclear about the current strength of evidence and the stability of risk estimates, and needed more information on how risk is calculated, more evidence that the benefits of reduced screening outweighed the potential harms, and a plan for reassessing risk at regular intervals (13 excerpts, four studies).^76,77,79,81^ Second, health-care professionals were concerned that some individuals could misinterpret risk scores or use low-risk results as a reason to disengage from screening (seven excerpts, four studies).^53,77,79,83^ Third, health-care professionals were concerned that reduced screening would lead to more interval cancers (six excerpts, three studies).^77–79^ Finally, health-care professionals raised questions about whether PRBS should be introduced to individuals who are already on the population-based screening programme or only to those new to screening (eight excerpts, two studies).^77,79^ They acknowledged that targeting individuals not yet screened for PRBS would be more feasible, but might lead to disparities in use of preventive care for other individuals.^77,79^

The pooled estimate of acceptability of PRBS among health-care professionals from seven studies was 86% (95% CI 64–99),^34,42–44,47,52,53^ which was not significantly different from acceptability among the general public (p=0·55; figure 2). One study found that 85% of health-care professionals thought it was important to move towards a PRBS programme.^33^ Similar to the general public, health-care professionals were more hesitant about lowering screening frequencies; from one study, 88% (85–90) intended to screen their patients more if they were at high risk, whereas 35% (31–39) intended to screen their patients less if they were at low risk.^45^ In four studies reporting on use of a PRBS tool, high proportions of health-care professionals (ranging from 79% to 100%) reported positive outcomes, such as the tool improving their practice, being easy to use, and facilitating screening decisions.^43,44,47,53^

There were 141 qualitative excerpts on barriers and facilitators to implementation of PRBS from 13 studies and 314 health-care professionals.^26,52,75–85^ At the individual level, health-care professionals frequently noted a lack of time and training as barriers. Explaining how to use the tool to patients, explaining risk results, and answering patients’ questions was time consuming and could be performed by nurses or other trained personnel instead (21 excerpts, eight studies).^26,75,76,78,80,81,83,85^ Health-care professionals noted that classifying risk, weighing different risk factors, incorporating their clinical judgement, and communicating this to the patient was complex, and providers would need training (12 excerpts, six studies).^26,76,80,83–85^ Health-care professionals recommended that risk assessment tools be well integrated into clinical workflows (eg, align with information already being collected) and not take attention away from the patient.^75,78,81,82,84^ They also reported that risk assessment tools could be helpful in facilitating conversations about cancer screening and that some patients might understand the importance of screening with an individualised risk score (six excerpts, two studies).^75,78^

In the quantitative studies, challenges in implementation of PRBS were also raised.^33,34,52,53,86^ Difficulty with communicating risk was also noted as a potential barrier in two studies.^33,34^ Six quantitative studies examined self-efficacy in the context of PRBS.^42,43,45,47,86,87^ There was varying confidence in engaging with PRBS tools and communicating results with studies reporting contradictory findings.^45,47,86^ Trust in PRBS among the general public and health-care professionals was also raised as an important facilitator.^33,34^ Health-care professionals believed that implementing PRBS should allow individuals to take a more active role in their care, expand access to screening, and optimise the benefits while minimising the harms of screening.^34,52,87^

Health-care professionals highlighted several implementation challenges at the system level, such as the need for policy support, funding, alignment with national guidelines, information on cost-effectiveness, and more personnel to handle risk assessments and the potential increase in screening volumes (38 excerpts, nine studies).^25,75–77,79,80,83–85^ Many health-care professionals highlighted the need for technology to support implementation of PRBS, including ensuring that individuals are placed on the correct pathway based on their risk, invited to screening at the right intervals, that information from electronic medical records are pulled correctly, and risk tools are integrated in the medical record (15 excerpts, seven studies).^52,75–78,80,84^ Some health-care professionals suggested a centralised screening programme with risk clinics (two excerpts, one study).^85^ Some health-care professionals pointed out that an ineffective risk-based screening programme could cause more anxiety to individuals than population-based screening and might exacerbate cancer screening disparities if individuals at high risk do not have access to resources to manage their risk or to be screened more often (three excerpts, two studies).^80,83^

Several system-level barriers and facilitators to implementing PRBS were also discussed in the quantitative studies. Across four studies reporting barriers faced by health-care professionals, the need for training was reported by 56% (95% CI 38–73) of health-care professionals and time constraints were reported by 44% (15–75) of health-care professionals (table).^33,34,42,86^ To facilitate the implementation of PRBS, health-care professionals reported that the volume of providers, access to resources such as full-time screening coordinators, quality improvement projects, and coordination of care are key factors that would affect the success of integrating PRBS paradigms.^33,45,52,88^

In subgroup analyses, among the qualitative findings, we did not find major differences by cancer site, publication date, or participants’ sex and age, but we did find differences by geographic region. Among the quantitative findings, we observed statistically significant differences in acceptability by cancer site for health-care professionals, and by cancer site, region, and sex for general public participants (appendix pp 53–56).

## Discussion

**Figure 3:**
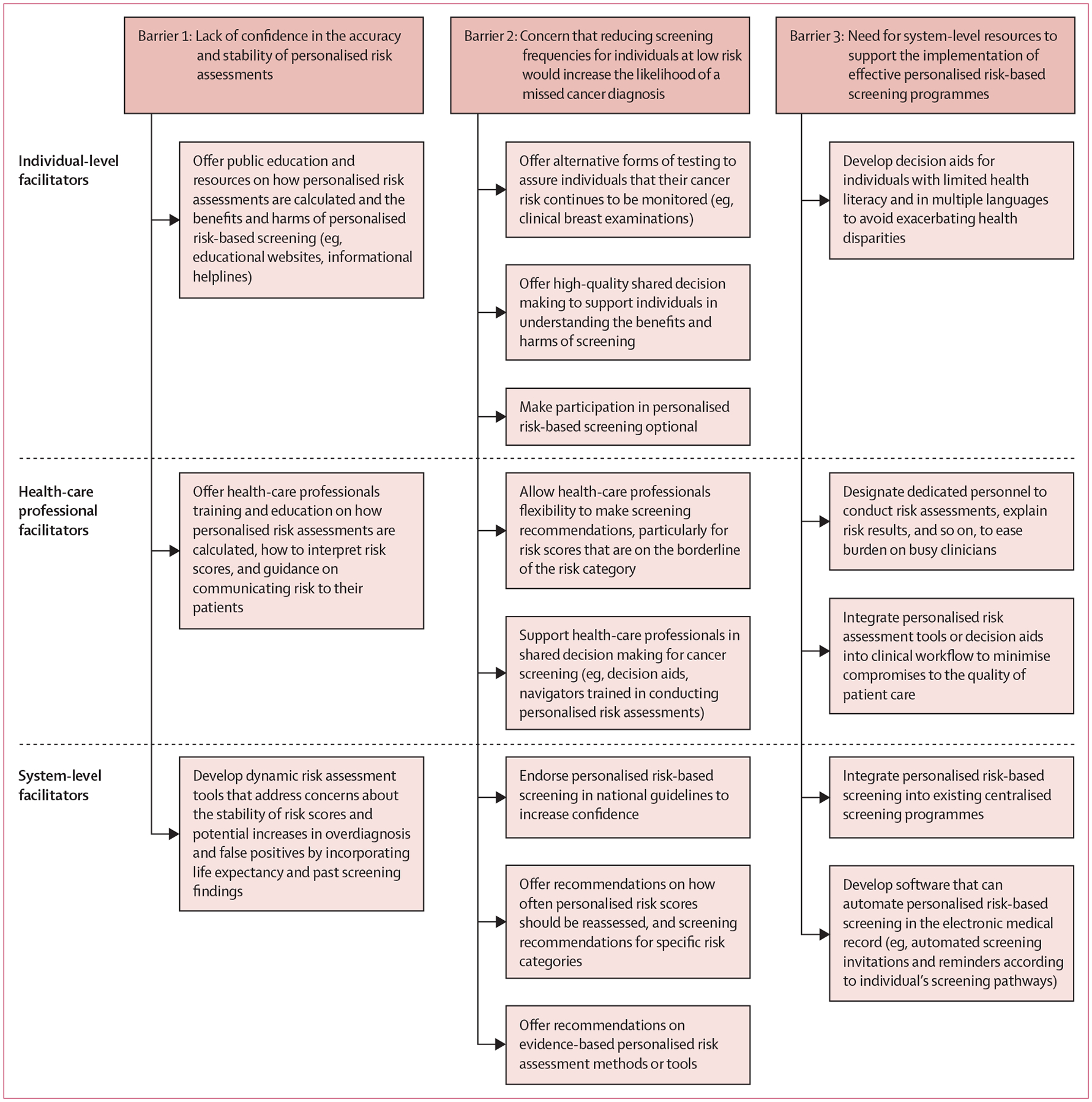
Summary of key barriers identified in the review and facilitators or recommendations at the individual, health-care professional, and system levels

To our knowledge, this systematic review is one of the few comprehensive and up-to-date reviews on this topic. We found that both the general public and health-care professionals find PRBS acceptable and view it as the next logical step in cancer early detection. An underlying message is that the shift towards a PRBS paradigm is an opportunity to enhance patient-centred care. The personalisation of cancer risk and the individual tailoring of screening pathways would provide individuals with tools to manage their risk or be proactive about cancer detection. Implementation of PRBS could potentially alleviate disparities arising from population-based guidelines^89^ and enhance health equity by opening up access to screening among individuals experiencing a greater burden of cancer. Finally, individuals will potentially have greater awareness of their options and more opportunity to be engaged in shared decision making. However, there remained concerns about PRBS, including a lack of knowledge of how PRAs were calculated, uncertainty about its accuracy and stability over time, and some participants expressed that PRAs might not change their risk perceptions. In the following paragraphs, we outline key barriers identified in our review and recommendations on facilitators to address these barriers (summarised in figure 3).

Confidence in PRAs among the general public and health-care professional needs to be improved to increase acceptability of PRBS. For example, there could be greater public education and resources (eg, educational websites and an informational helpline) to improve knowledge about how PRAs are calculated and the potential benefits and harms of PRBS. Evaluations of online risk calculators have found that few provide information on the statistical model used or information to assess the validity of risk estimates, suggesting that individuals are not routinely educated about how risk is calculated.^90^ Personalised risk scores should also be interpreted in the context of other information that might influence individual risk perception, such as knowledge of risk factors, family health history, self-reported health status, and social comparisons with peers, to minimise scepticism about risk results.^91,92^ Future interventions should also focus on educating health-care professionals on how risk scores are calculated and how to interpret and communicate risk. At the system level, future research can help to develop risk assessment tools that address the concerns of individuals and health-care professionals without compromising the effectiveness of screening. Dynamic risk assessment tools, such as the ENGAGE framework, can address concerns about the stability of risk scores over time and potential increases in overdiagnosis and false positives by incorporating life expectancy and past screening findings.^93^

We found that general public participants and health-care professionals in our review were primarily concerned about the reduction in screening frequencies for individuals at low risk. To reassure such individuals, alternative forms of testing, such as clinical breast examinations or multi-cancer early detection tests could be offered, as long as they do not compromise the efficiency and cost-effectiveness of screening programmes. The acceptability of PRBS could also be improved by making participation optional, or by varying age at which screening starts based on risk level, such as for breast cancer screening.^94^ Additionally, facilitating high-quality shared decision-making conversations about the potential benefits and harms of each option is critical. Studies have found that health-care professionals tend to emphasise screening benefits;^95^ it is crucial to provide all individuals, particularly those at low risk, with information about screening harms to understand why they might benefit from reducing their screening frequency. For health-care professionals, allowing flexibility to make screening recommendations is essential, particularly for individuals on the borderline of risk categories (eg, a risk score that is on the high end of the low-risk category). Health-care professionals can also be supported in conducting shared decision making about cancer screening (eg, decision aids or navigators trained in conducting PRAs). Finally, at the system level, the acceptability of PRBS could be improved through its endorsement in national guidelines (eg, US Preventive Services Task Force), providing recommendations on evidence-based PRA methods or tools, recommendations on how often PRA should be reassessed, and screening or surveillance recommendations for specific risk categories.

Finally, dedicated personnel to conduct PRAs, explain risk results, answer concerns, and provide recommendations on risk management are a system-level facilitator to implementing PRBS. Dedicated and trained personnel could be integrated into existing centralised cancer screening programmes, some of which might already be performing PRAs.^96^ To effectively support health-care professionals in performing PRAs, risk stratification tools or decision aids need to be integrated into the clinical workflow and facilitate conversations without compromising the quality of patient care. Availability of these resources would address the barrier of time constraints among busy health-care professionals. Future research should also focus on developing tools in multiple languages and for individuals with limited health literacy to avoid exacerbating health disparities. Studies have found that the majority of breast cancer risk assessment tools on the internet scored low on readability, and that individuals with a lower education level and who were from minority ethnic groups were less likely to accurately repeat back their risk estimate, suggesting lower comprehension of risk results.^39,97^ Finally, as frequently reported by health-care professionals in this review, electronic medical record-integrated software that can perform PRAs, automate screening invitations and reminders according to the individual’s screening pathway, and consolidate medical history and screening results, is crucial to the successful implementation of PRBS.

The comprehensiveness and breadth of studies we analysed are a key strength of our review but also resulted in heterogeneity between studies. Overall, our subgroup analysis of the qualitative data did not find major divergences from the main results. Although the majority of the studies were from North America and Europe and were about breast cancer screening, similar findings were found across the other subgroups, albeit in lower frequencies. We observed willingness to engage in PRA and satisfaction with PRBS to be higher among younger participants (aged <50 years), perhaps because many have not started screening yet and are more open to new screening technologies. Of note, we observed that North American participants were less likely to want to screen less if at low risk and screen more if at high risk, compared with their European counterparts. A possible explanation for this finding is concerns about health-care costs among US participants, who might be concerned about missing a cancer diagnosis if screened less and about additional out-of-pocket costs if screened more.

Our findings were consistent with previously published reviews^19,20^ and existing reviews about implementation of risk-based cancer screening,^20,98^ but our systematic review includes an additional 42 studies and evaluates a broader scope of outcomes. Previous reviews had examined only acceptability, whereas our review assessed willingness to engage in PRA, satisfaction with PRBS, adherence to recommended screening protocols, and behavioural modifications based on PRAs. Additionally, we conducted a meta-analysis of eight quantitative outcomes, conducted subgroup analyses, and integrated the qualitative and quantitative findings to give a holistic view of the state of the evidence. In addition, our review synthesises perceptions and acceptability of PRBS among both the general public and health-care professionals, identifies areas of concern and barriers, and provides recommendations for facilitators at the individual, health-care professional, and system level to support uptake of a PRBS paradigm.

This study had some limitations. First, most studies included in the systematic review were about breast cancer screening and findings on cervical and prostate cancer screening were from the general public only. More research on PRBS for colorectal, lung, prostate, and cervical cancer screenings is needed to understand the specific needs for PRBS in those screening contexts. Future research should also focus on the acceptability and perceptions of PRBS among health-care professionals, because successful implementation will be dependent on health-care professionals performing PRAs and recommending PRBS to their patients. Second, our quantitative analysis on behavioural modifications based on PRAs focused on participants who were categorised as high or low risk, because these categories were the most commonly reported across included studies. Little is known about how receiving an average risk score would affect acceptability of PRAs, PRBS, and screening intentions or behaviour, a potential area for future research. Third, some of the pooled estimates in the meta-analysis included few studies. Cautious interpretation is needed in these cases. Finally, because PRBS is not yet part of routine clinical practice, we included studies with both actual and hypothetical PRAs and risk assessment tools or scenarios. We interpreted the findings from both actual and hypothetical studies in the same way and did not perform a subgroup analysis. As PRBS becomes more widely used, future studies might wish to focus on actual PRAs and risk assessment tools.

In conclusion, in principle, the general public and health-care professionals both viewed PRAs as providing valuable information and PRBS as a logical next step to increase the quality of patient care and improve cancer mortality. Steps to address concerns related to educating individuals and providers on PRAs, time constraints, technology, and policy support are warranted to ensure the successful implementation of PRBS.

## Supplementary Material

1

## Figures and Tables

**Table:** Acceptability, satisfaction, and implementation outcomes among general public participants and health-care professionals in the included studies

	Number of studies	Sample size	Percentage (95% CI)	*I* ^2^
**Acceptability**				
General public participants	13*	17 446	77·91% (65·62–88·13)	99·67%
Health-care professionals	7	292	86·28% (63·79–99·45)	93·02%
Pooled	20	17 738	80·42% (69·91–89·21)	99·54%†
**Willingness to engage in PRA**				
General public participants	5*	9746	84·26% (70·93–94·08)	99·52%
**Satisfaction with PRBS**				
General public participants	3	242	90·49% (76·35–98·86)	87·06%
**Intention to screen more if high risk**				
General public participants	8*	14 410	85·99% (73·44–95·04)	99·68%
Health-care professionals	1	593	87·69% (84·92–90·22)	NA
Pooled	9	15 003	86·13% (74·87–94·47)	99·63%†
**Intention to screen less if low risk**				
General public participants	9*	14 537	56·46% (46·12–66·52)	99·26%
Health-care professionals	1	593	35·08% (31·28–38·97)	NA
Pooled	8	14 537	54·64% (44·65–64·44)	99·24%‡
**Intended adherence (to recommended screening protocols)**				
General public participants	2	485	30·90% (0·00–94·30)	99·57%
**Training required**				
Health-care professionals	4	986	55·93% (37·89–73·22)	95·74%
**Time constraints**				
Health-care professionals	4	985	43·57% (14·54–75·20)	98·74%

NA=not applicable. PRA=personalised risk assessment. PRBS=personalised risk-based screening.

* For Koitsalu et al,^51^ the study population was split into men and women because data were provided for each cohort.

† p value for subgroup differences >0·05.

‡ p value for subgroup differences=0·0001.

## Data Availability

Data for this study are available in the appendix or upon reasonable request made to the corresponding author.

## Research in context

### Evidence before this study

We searched PubMed for reviews of personalised risk-based screening (PRBS) for cancer published up to Aug 30, 2022, when the PROSPERO protocol for this study was registered. We limited our search to English language publications and used the search terms: (risk-based screening OR personalised risk assessment OR risk calculator) AND (cancer screening) AND (review). We identified 1594 publications. Most reviews on this topic were focused on specific cancers. We identified two reviews on acceptability of PRBS for multiple cancers, but the findings for the general public and health-care professionals were interpreted and published separately.

### Added value of this study

In this systematic review and meta-analysis, we present a comprehensive overview of the acceptability and perceptions of PRBS among the general public and health-care professionals for five cancer sites with established screening programmes (ie, breast, lung, colorectal, cervical, and prostate cancer screening). Compared with previous reviews, this study assesses a broader scope of outcomes beyond acceptability, including willingness to engage in personalised risk assessment, satisfaction, adherence to recommended screening protocols, intentions to screen more or less based on risk results, and barriers for health-care professionals (eg, training required and time constraints). Additionally, our review includes a total of 63 studies, 42 additional studies compared with the two aforementioned reviews, and includes a meta-analysis to provide pooled estimates for the quantitative outcomes extracted from the studies. Based on our findings, we also provide recommendations at the public, health-care professional, and system level to support the implementation of PRBS in routine clinical practice.

### Implications of all the available evidence

Our findings indicate that both the general public and health-care professionals find PRBS acceptable and view it as the next logical step in cancer prevention and control. An underlying message is that the shift towards a PRBS paradigm is an opportunity to enhance patient-centred care, mitigate health inequity by improving access to screening, and empower the general public to become more engaged in decision making. However, concerns among the general public and health-care professionals about the accuracy of personalised risk assessment and its stability over time, the potential of interval cancers among individuals deemed to be at low risk who might be screened less, and the resources needed to successfully implement PRBS in busy clinical settings need to be addressed before PRBS can become part of routine practice.
