# Supplementary material for: Acceptability and perceptions of personalised risk-based cancer screening among health-care professionals and the general public: a systematic review and meta-analysis
Source: Lancet Public Health. Author manuscript; Available in PMC 2025 Feb 12. (PMC11817692; doi:10.1016/S2468-2667(24)00278-0)
Supplement: 1 [file NIHMS2054260-supplement-1.pdf]

THE LANCET
Public Health

Supplementary appendix

This appendix formed part of the original submission and has been peer reviewed. We post it as supplied by the authors.

Supplement to: Tan NQP, Nargund RS, Douglas EE, et al. Acceptability and perceptions of personalised risk-based cancer screening among health-care professionals and the general public: a systematic review and meta-analysis. *Lancet Public Health* 2025; **10:** e85–96.

Appendix

Table of Contents

Appendix A: Search strategy. 2

Appendix B: Data extraction forms and risk of bias tool. 5

Appendix C: Subgroup analysis methods 8

Appendix D: Reference list of articles excluded at the full-text screening stage. 9

Appendix E: Characteristics of included studies (N=63). 14

Appendix F: Characteristics of participants. 25

Appendix G: Quality appraisal scores of included studies (N=63). 33

Appendix H: Summary of themes extracted from qualitative and mixed method studies. 37

Appendix I: Findings extracted from quantitative studies and mixed method studies. 39

Appendix J: Themes identified from qualitative (n=22) and mixed methods studies (n=10) and supporting quotations. 46

Appendix K: Additional meta-analysis results. 49

Appendix L: Subgroup analyses results. 53

Appendix A: Search strategy.

**Database**	**Search strings**
Ovid MEDLINE	1. "Early Detection of Cancer"/<br>2. Early Diagnosis/<br>3. (cancer and screen*).ti.<br>4. ((cancer ADJ3 screen*) or (personal* adj2 screen*)).ti,ab.<br>5. OR/1-4<br>6. exp Breast Neoplasms/<br>7. exp Uterine Cervical Neoplasms/<br>8. exp Colorectal Neoplasms/<br>9. exp Lung Neoplasms/<br>10. exp Prostatic Neoplasms/<br>11. ((breast or cervical or cervix or colon or colorectal or "colo-rectal" or lung or prostat* or rectal) ADJ2 (adenocarcinoma* or cancer* or carcinoma* or neoplas* or sarcoma*)).ti,ab.<br>12. OR/6-11<br>13. Risk Management/<br>14. Risk Assessment/<br>15. Risk Factors/<br>16. (risk ADJ2 (analysis or assess* or based or calculat* or cancer or estimat* or evaluat* or individual* or manag* or model* or personal* or prediction or stratifi* or tailor* or tool) or (predictive ADJ2 model*)).ti,ab.<br>17. OR/13-16<br>18. Patient Satisfaction/<br>19. Patient Preference/<br>20. Trust/<br>21. Health Services Accessibility/<br>22. ((patient* or women* or men or men's) ADJ2 (accept* or attitude* or access* or barrier* or confidence or insight* or perceive* or perception* or perspective* or prefer* or satisf* or trust)).ti,ab<br>23. OR/18-22<br>24. Exp Health Personnel/<br>25. Attitude of Health Personnel/<br>26. (doctor* or gastroenterologist* or gynecologist* or "health care provider*" or nurse? or oncologist* or physician* or proctologist* or pulmonologist* or radiologist* or ((health or healthcare) ADJ1 (personnel or provider* or practitioner*) ADJ2 (accept* or attitude* or access* or barrier* or confidence or insight* or perceive* or perception* or perspective* or prefer* or satisf* or trust))).ti,ab.<br>27. OR/24-26<br>28. OR/23,27<br>29. AND/5,12,17,28<br>30. Limit 29 to English language<br>31. limit 30 to yr="2010-current" [retrieves 1378]
Ovid Embase	1. Cancer Screening/<br>2. (cancer and screen*).ti.<br>3. ((cancer ADJ3 screen*) or (personal* adj2 screen*)).ti,ab.<br>4. OR/1-3<br>5. exp Breast Cancer/<br>6. exp Uterine Cervix Cancer/<br>7. exp Colorectal Cancer/<br>8. exp Lung Cancer/<br>9. exp Prostate Cancer/<br>10. ((breast or cervical or cervix or colon or colorectal or "colo-rectal" or lung or prostat* or rectal) ADJ2 (adenocarcinoma* or cancer* or carcinoma* or neoplas* or sarcoma*)).ti,ab.<br>11. OR/5-10<br>12. Cancer Risk/<br>13. High Risk Population/<br>14. High Risk Patient/<br>15. Patient Risk/<br>16. Predictive Model/<br>17. Risk Algorithm/<br>18. Exp Risk Assessment/<br>19. Risk Benefit Analysis/<br>20. Risk Factor/<br>21. Risk Model/<br>22. Risk Perception/<br>23. (risk ADJ2 (analysis or assess* or based or calculat* or cancer or estimat* or evaluat* or individual* or manag* or model* or personal* or prediction or stratifi* or tailor* or tool) or (predictive ADJ2 model*)).ti,ab.

	24. OR/12-23<br>25. Health Care Access/<br>26. Patient Satisfaction/<br>27. Patient Attitude/<br>28. Patient Preference/<br>29. Perception/<br>30. ((patient* or women* or men or men's) ADJ2 (accept* or attitude* or access* or barrier* or confidence or insight* or perceive* or perception* or perspective* or prefer* or satisf* or trust)).ti,ab.<br>31. OR/25-30<br>32. Exp Physician/<br>33. Physician Attitude/<br>34. ((doctor* or gastroenterologist* or gynecologist* or ((health or healthcare or "health care") ADJ1 (personnel or provider* or practitioner*)) or nurse? or oncologist* or physician* or proctologist* or pulmonologist* or radiologist*)) adj2 (accept* or attitude* or access* or barrier* or confidence or insight* or perceive* or perception* or perspective* or prefer* or satisf* or trust).ti,ab.<br>35. OR/32-34<br>36. OR/31,35<br>37. AND/4,11,24,36<br>38. Limit 37 to English language<br>39. limit 38 to yr="2010-current" [retrieves 2872]
Cochrane Central	cancer NEXT/3 screen* in Title Abstract Keyword AND (breast or cervical or cervix or colon or colorectal or "colo-rectal" or lung or prostat* or rectal) NEXT/3 (adenocarcinoma* or cancer* or carcinoma* or neoplas* or sarcoma*) in Title Abstract Keyword AND (risk NEXT/1 (analysis or assess* or based or calculat* or cancer or estimat* or evaluat* or individual* or manag* or model* or personal* or prediction or stratifi* or tailor* or tool)) or (predictive NEAR/2 model*) in Record Title AND accept* or attitude* or access* or confidence or insight* or perceive* or perception* or perspective* or prefer* or satisf* or trust in Title Abstract Keyword - (Word variations have been searched)
Ovid PsycINFO	1. "Cancer Screening"/<br>2. (cancer and screen*).ti.<br>3. ((cancer adj3 screen*) or (personal* adj2 screen*)).ti,ab.<br>4. or/1-3<br>5. exp Breast Neoplasms/<br>6. ((breast or cervical or cervix or colon or colorectal or "colo-rectal" or lung or prostat* or rectal) adj2 (adenocarcinoma* or cancer* or carcinoma* or neoplas* or sarcoma*)).ti,ab.<br>7. OR/5-6<br>8. Risk Assessment/<br>9. ((risk adj2 (analysis or assess* or based or calculat* or cancer or estimat* or evaluat* or individual* or manag* or model* or personal* or prediction or stratifi* or tailor* or tool)) or (predictive adj2 model*)).ti,ab.<br>10. OR/8-9<br>11. Patient Satisfaction/<br>12. Trust/<br>13. ((patient* or women* or men or men's) adj2 (accept* or attitude* or access* or barrier* or confidence or insight* or perceive* or perception* or perspective* or prefer* or satisf* or trust)).ti,ab.<br>14. exp Health Personnel/<br>15. Health Personnel Attitudes/<br>16. (doctor* or gastroenterologist* or gynecologist* or "health care provider*" or nurse? or oncologist* or physician* or proctologist* or pulmonologist* or radiologist* or ((health or healthcare) adj1 (personnel or provider* or practitioner*) adj2 (accept* or attitude* or access* or barrier* or confidence or insight* or perceive* or perception* or perspective* or prefer* or satisf* or trust))).ti,ab.<br>17. OR/11-16<br>18. AND/4,7,10,17<br>19. Limit 18 to English language<br>20. limit 19 to yr="2010-current" [retrieves 106]
EBSCOhost CINAHL	S1 AND S8 AND S11 AND S12<br>S12 ((MH "Patient Attitudes") OR (MH "Attitude of Health Personnel+") OR (MH "Physician Attitudes") OR (MH "Nurse Attitudes") OR (MH "Attitude to Risk") OR (MH "Attitude to Medical Treatment") OR (MH "Attitude Measures") OR (MH "Patient Satisfaction+") OR (MH "Patient Preference") OR (MH "Family Attitudes+") OR (MH "Personal Satisfaction+")) OR TI (((patient* or women* or men or men's) W2 (accept* or attitude* or access* or barrier* or confidence or insight* or perceive* or perception* or perspec ...<br>S11 S9 OR S10<br>S10 TI ((risk W2 (analysis or assess* or based or calculat* or cancer or estimat* or evaluat* or individual* or manag* or model* or personal* or prediction or stratifi*) or (predictive W2 model*))) OR AB ((risk W2 (analysis or assess* or based or calculat* or cancer or estimat* or evaluat* or individual* or manag* or model* or personal* or prediction or stratifi*) or (predictive W2 model*)))<br>S9 (MH "Risk Assessment") OR (MH "Attitude to Risk")<br>S8 S2 OR S3 OR S4 OR S5 OR S6 OR S7<br>S7 TI (((breast or cervical or cervix or colon or colorectal or "colo-rectal" or lung or prostat* or rectal) W2 (adenocarcinoma* or cancer* or carcinoma* or neoplas* or sarcoma*))) OR AB (((breast or cervical or cervix or colon or

	colorectal or "colo-rectal" or lung or prostat* or rectal) W2 (adenocarcinoma* or cancer* or carcinoma* or neoplas* or sarcoma*)))<br>S6 (MH "Prostatic Neoplasms+")<br>S5 (MH "Lung Neoplasms+")<br>S4 (MH "Colorectal Neoplasms+")<br>S3 (MH "Cervix Neoplasms+")<br>S2 (MH "Breast Neoplasms+")<br>S1 (MH "Cancer Screening") OR TI ((cancer and screen*)) OR (((cancer W3 screen*) or (personal* W3 screen*))) OR (AB (((cancer and screen*)) OR (((cancer W3 screen*) or (personal* W3 screen*)))))

Appendix B: Data extraction forms and risk of bias tool.

**Data Extraction Form**	
**Study Characteristics**	
First author's last name (publication year)	___________________
Study funding sources	___________________
Article type	o Full text article<br>o Abstract
Study design	o Randomized controlled trial<br>o Non-randomized experimental study<br>o Cohort study<br>o Cross-sectional study<br>o Case-control study<br>o Before-and-after study<br>o Qualitative research<br>o Other: ________________
Screening context	o Breast cancer screening<br>o Cervical cancer screening<br>o Prostate cancer screening<br>o Lung cancer screening<br>o Colorectal cancer screening
Population	o Health care providers or professionals<br>o Patients or participants
Country in which the study was conducted	o United States<br>o UK<br>o Canada<br>o Australia<br>o Netherlands<br>o Spain<br>o Other: ________________
Study setting	o Primary care clinic<br>o Hospital<br>o Online (web-based)<br>o Community clinic<br>o VA system<br>o Participants of a previous trial<br>o Other: ________________
Number of centers (recruitment)	o Single center study<br>o Multi-center study<br>o NA, not clinical setting<br>o Other: ________________
Inclusion criteria	___________________
Data collection period	___________________
Data collection method	o Survey<br>o Interview<br>o Focus group<br>o Other: ________________
Follow-up assessments	___________________
**Intervention Characteristics**	
Risk assessment tool/method used	___________________
Description of how the risk assessment tool was used (if applicable)	___________________
Were participants given their risk score?	o Yes<br>o No<br>o NA, hypothetical<br>o NA, participant is not a patient
**Participant Characteristics**	
Sample size	___________________
Race and ethnicity	___________________
Gender	___________________

Age	___________________
Education level	___________________
Income level or socioeconomic status	___________________
Insurance status	___________________
Specialty (if HCP)	___________________
Level of experience (if HCP)	___________________
Patient volume (if HCP)	___________________
Quantitative outcomes	___________________

Mixed Methods Appraisal Tool (MMAT)		
**Category of study designs**	**Methodological quality criteria**	**Responses**
Screening questions (for all types)	S1. Are there clear research questions?	Yes/No/Can't tell
	S2. Do the collected data allow to address the research questions?	Yes/No/Can't tell
	Further appraisal may not be feasible or appropriate when the answer is 'No' or 'Can't tell' to one or both screening questions.	
1. Qualitative	1.1. Is the qualitative approach appropriate to answer the research question?	Yes/No/Can't tell
	1.2. Are the qualitative data collection methods adequate to address the research question?	Yes/No/Can't tell
	1.3. Are the findings adequately derived from the data?	Yes/No/Can't tell
	1.4. Is the interpretation of results sufficiently substantiated by data?	Yes/No/Can't tell
	1.5. Is there coherence between qualitative data sources, collection, analysis, and interpretation?	Yes/No/Can't tell
2. Quantitative randomized controlled trials	2.1. Is randomization appropriately performed?	Yes/No/Can't tell
	2.2. Are the groups comparable at baseline?	Yes/No/Can't tell
	2.3. Are there complete outcome data?	Yes/No/Can't tell
	2.4. Are outcome assessors blinded to the intervention provided?	Yes/No/Can't tell
	2.5. Did the participants adhere to the assigned intervention?	Yes/No/Can't tell
3. Quantitative non-randomized	3.1. Are the participants representative of the target population?	Yes/No/Can't tell
	3.2. Are measurements appropriate regarding both the outcome and intervention (or exposure)?	Yes/No/Can't tell
	3.3. Are there complete outcome data?	Yes/No/Can't tell
	3.4. Are the confounders accounted for in the design and analysis?	Yes/No/Can't tell
	3.5. During the study period, is the intervention administered (or exposure occurred) as intended?	Yes/No/Can't tell
4. Quantitative descriptive	4.1. Is the sampling strategy relevant to address the research question?	Yes/No/Can't tell
	4.2. Is the sample representative of the target population?	Yes/No/Can't tell
	4.3. Are the measurements appropriate?	Yes/No/Can't tell
	4.4. Is the risk of nonresponse bias low?	Yes/No/Can't tell
	4.5. Is the statistical analysis appropriate to answer the research question?	Yes/No/Can't tell
5. Mixed methods	5.1. Is there an adequate rationale for using a mixed methods design to address the research question?	Yes/No/Can't tell
	5.2. Are the different components of the study effectively integrated to answer the research question?	Yes/No/Can't tell
	5.3. Are the outputs of the integration of qualitative and quantitative components adequately interpretated?	Yes/No/Can't tell
	5.4. Are divergences and inconsistencies between quantitative and qualitative results adequately addressed?	Yes/No/Can't tell
	5.5. Do the different components of the study adhere to the quality criteria of each tradition of the methods involved?	Yes/No/Can't tell

Appendix C: Subgroup analysis methods.

Subgroup Analyses Methods

We conducted subgroup analysis by cancer site (breast, cervical, colorectal, lung, and prostate) as planned in the protocol. After reviewing the data, we also conducted subgroup analysis by geographic region (North America, Europe, Asia Pacific, and West Pacific), publication year (2010-2018 or 2019-2024), sex of participants, and age of participants on both the qualitative and quantitative findings. We performed meta-regressions to determine if the year of publication and age of the participants, as continuous variables, impacted the overall effect size of the main outcomes. For qualitative findings, we examined the excerpts, initial codes, and descriptive codes within each topic and compared them across the subgroups to identify any divergences from our main results.

Appendix D: Reference list of articles excluded at the full-text screening stage.

1. ACTRN. The CRISP (Colorectal cancer RISk Prediction tool) trial: providing personalised advice about bowel cancer screening in primary care. 2015; http://wwwwhoint/trialsearch/Trial2aspx?TrialID=ACTRN12615000155594 [link unavailable].
2. Ali N, Lifford KJ, Carter B, et al. Barriers to uptake among high-risk individuals declining participation in lung cancer screening: a mixed methods analysis of the UK lung cancer screening (UKLS) trial. BMJ Open. 2015;5(7). https://dx.doi.org/10.1136/bmjopen-2015-008254
3. Allen JD, Filson CP, Berry DL. Effect of a prostate cancer screening decision aid for African-American men in primary care settings. Cancer Epidemiol Biomarkers. 2020;29(11):2157-2164. https://dx.doi.org/10.1158/1055-9965.EPI-20-0454
4. Aminololama-Shakeri S, Soo MS, Grimm LJ, Destounis S. Screening guidelines and supplemental screening tools: assessment of the adequacy of patient-provider discussions. J Breast Imaging. 2019;1(2):109-114. https://dx.doi.org/10.1093/jbi/wbz019
5. American Family Physician. Screening for prostate cancer: recommendation statement. 2018;98(8):536A-536G.
6. Bancroft EK, Castro E, Page E, et al. The PROFILE study; Germline genetic profiling: Correlation with targeted prostate cancer screening and treatment. Cancer Research Conference: 103rd Annual Meeting of the American Association for Cancer Research, AACR. 2012;72(8 Supp 1). https://dx.doi.org/10.1158/1538-7445.AM2012-2612
7. Beyer K, Leenen R, Venderbos LDF, et al. Understanding the barriers to prostate cancer population-based early detection programs: The PRAISE-U BEST survey. European Urology. 2024;85(Supp 1):S1612. https://dx.doi.org/10.1016/S0302-2838%2824%2901238-7
8. Bidassie B, Kovach A, Vallette MA, Merriman J, Park YA, Aggarwal A, Colonna S. Breast cancer risk assessment and chemoprevention use among veterans affairs primary care providers: a national online survey. Mil Med. 2020;185(3-4):512-518. https://dx.doi.org/10.1093/milmed/usz291
9. Brooks R, Saya S, Emery J. Patients' views and experiences of genomic testing for cancer risk prediction: A qualitative sub-study of the SCRIPT trial. Aust J Prim Health. 2023;29(4):xiv. https://dx.doi.org/10.1071/PYv29n4abs
10. Burke C, Leach B, Dai J, et al. Community uptake of an online CRC risk assessment. Am J Gastroenterol. 2010;1:S549. https://dx.doi.org/10.1038/ajg.2010.320-13
11. Cao P, Caverly T, Hayward R, Meza R. Effect of cancer risk and patient preferences on net benefit of lung cancer screening: a personalized lung cancer screening model. Cancer Epidemiol Biomarkers Conference: AACR Conference on the Improving Cancer Risk Prediction for Prevention and Early Detection Orlando, FL United States. 2017;26(5 Supp 1). https://dx.doi.org/10.1158/1538-7755.CARISK16-PR16
12. Carlsson SV, Preston MA, Vickers A, Malhotra D, Ehdaie B, Healey MJ, Kibel AS. A provider-facing decision support tool for prostate cancer screening in primary care: a pilot study. Appl Clin Inform. 2024;15(2):274-281. https://dx.doi.org/10.1055/s-0044-1780511
13. Chouhayd El Ataoui L, Lopez-Castellano AC, Castillo Garcia E. Implementation and evaluation of a breast cancer pharmaceutical advisory programme. Pharmaceutical Care Espana. 2014;16(4):57.
14. Cremers R, Van Asperen C, Kil P, Vasen H, Wiersma T, Van Oort I, Kiemeney L. Urologists' and GPs' knowledge of hereditary prostate cancer is suboptimal for prostate cancer counseling: a nation-wide survey in The Netherlands. Fam Cancer. 2012;11(2):195-200. https://dx.doi.org/10.1007/s10689-011-9500-8
15. da Rocha Araujo FAG, Barroso UDO. Prostate cancer screening: beliefs and practices of the Brazilian physicians with different specialties. J Eval Clin Pract. 2018;24(3):508-513. https://dx.doi.org/10.1111/jep.12901
16. Dwyer AA, Hesse-Biber S, Flynn B, Remick S. Parent of origin effects on family communication of risk in BRCA+ women: a qualitative investigation of human factors in cascade screening. Cancers. 2020;12(8):1-16. https://dx.doi.org/10.3390/cancers12082316
17. Eden KB, Ivlev I, Bensching KL, et al. Use of an online breast cancer risk assessment and patient decision aid in primary care practices. J Womens Health. 2020;29(6):763-769. https://dx.doi.org/10.1089/jwh.2019.8143

18. Fisher BA, Wilkinson L, Valencia A. Women's interest in a personal breast cancer risk assessment and lifestyle advice at NHS mammography screening. J Public Health. 2017;39(1):113-121. doi:10.1093/pubmed/fdv211
19. Gin VN, Selvan P, Mohanty S, Lum R, Serrao S, Leader AE. Exploring Asian Indian and Pakistani views about cancer and participation in research: an evaluation of a culturally tailored educational intervention. Cancer Epidemiology Biomarkers and Prevention Conference: 11th AACR Conference on the Science of Cancer Health Disparities in Racial/Ethnic Minorities and the Medically Underserved New Orleans, LA United States 2020;29(6 Supp 1). https://dx.doi.org/10.1158/1538-7755.DISP18-A028
20. Gladysz K, Borella L. Early outcomes of abbreviated breast MRI for breast cancer screening in clinical practice. J Med Imaging Radiat Oncol. 2021;65(Supp 1):109-110. https://dx.doi.org/10.1111/1754-9485.13301
21. Green T, Martins T, Hamilton W, Rubin G, Elliott K, Macleod U. Exploring GPs' experiences of using diagnostic tools for cancer: a qualitative study in primary care. Fam Pract. 2015;32(1):101-5. https://dx.doi.org/10.1093/fampra/cmu081
22. Head M, Cohn B, Wernli KJ, Palazzo L, Ehrlich K, Matson A, Knerr S. Young women's perspectives on being screened for hereditary breast and ovarian cancer risk during routine primary care. Womens Health Issues. 2024. https://dx.doi.org/10.1016/j.whi.2024.01.004
23. Hindmarch S, Howell SJ, Usher-Smith JA, Gorman L, Evans DG, French DP. Feasibility and acceptability of offering breast cancer risk assessment to general population women aged 30-39 years: a mixed-methods study protocol. BMJ Open. 2024;14(1). https://dx.doi.org/10.1136/bmjopen-2023-078555
24. Iz FB, Tumer A. Assessment of breast cancer risk and belief in breast cancer screening among the primary healthcare nurses. J Cancer Educ. 2016;31(3):575-81. https://dx.doi.org/10.1007/s13187-015-0977-y
25. Jimbo M, Sen A, Plegue MA, et al. Interactivity in a decision aid: findings from a decision aid to technologically enhance shared decision making RCT. Am J Prev Med. 2019;57(1):77-86. https://dx.doi.org/10.1016/j.amepre.2019.03.004
26. Katz LH, Advani S, Burton-Chase AM, et al. Cancer screening behaviors and risk perceptions among family members of colorectal cancer patients with unexplained mismatch repair deficiency. Fam Cancer. 2017;16(2):231-237. https://dx.doi.org/10.1007/s10689-016-9947-8
27. Kerman BJ, Brunette CA, Harris EJ, Antwi AA, Lemke AA, Vassy JL. Primary care physician use of patient race and polygenic risk scores in medical decision-making. Genet Med. 2023;25(4):100800. https://dx.doi.org/10.1016/j.gim.2023.100800
28. Koitsalu M, Czene K, Sprangers M, Brandberg Y. Population survey on Swedish women's attitudes towards tailored mammography screening based on individual risk for breast cancer. Asia Pac J Clin Oncol. 2012;3:238. https://dx.doi.org/10.1111/ajco.12030
29. Kummer S, Waller J, Ruparel M, Cass J, Janes SM, Quaife SL. Mapping the spectrum of psychological and behavioural responses to low-dose CT lung cancer screening offered within a Lung Health Check. Health Expect. 2020;23(2):433-441. doi:10.1111/hex.13030
30. Lapointe J, Cote JM, Mbuya-Bienge C, et al. Attitudes and views of healthcare professionals on a risk-stratified breast cancer screening approach: A pan-Canadian cross-sectional survey. Fam Cancer. 2023;22(3):388. https://dx.doi.org/10.1007/s10689-023-00332-5
31. Larkin L, Magnante A. Changes in self-perceived breast cancer risk, risk factor awareness and risk-reducing behaviors after a breast cancer education session and individualized risk assessment. Menopause. 2019;26(12):1458. https://dx.doi.org/10.1097/GME.0000000000001456
32. Lee KWM, Chan SYS, Chan ESY, Hou SSM, Ng CF. To review the presentation pattern of newly diagnosed prostate cancer managed in a regional hospital. BJU International. 2014;1:7. https://dx.doi.org/10.1111/bju.12606
33. Leonarczyk TJ, Mawn BE. Cancer risk management decision making for BRCA+ women. West J Nurs Res. 2015;37(1):66-84. doi:10.1177/0193945913519870
34. Lieberman S, Lahad A, Tomer A, Cohen C, Levy-Lahad E, Raz A. Population screening for BRCA1/BRCA2 mutations: lessons from qualitative analysis of the screening experience. Genet Med. 2017;19(6):628-634. https://dx.doi.org/10.1038/gim.2016.175
35. Lippey J, Keogh L, Mann GB, Campbell I, Forrest L. Are Victorian women interested in risk stratified breast screening? Breast. 2020;50:153-154. https://dx.doi.org/10.1016/j.breast.2020.01.011

36. MacEachern J, Mathews M, Green J, Pullman D. Specialists' perceptions of hereditary colorectal cancer screening in Newfoundland and Labrador. Curr Oncol. 2012;19(3):e123-e128. https://dx.doi.org/10.3747/co.19.932
37. Maschke A, Battaglia TA, Schonberg MA, Paasche-Orlow M, Kressin NR, Gunn C. Risk and potential mammography harms discussion in breast cancer counseling: perspectives from patients with low health literacy and primary care providers. J Gen Intern Med. 2020;35(Supp 1):S260. https://dx.doi.org/10.1007/s11606-020-05890-3
38. Myers RE, Daskalakis C, Kunkel EJ, Cocroft JR, Riggio JM, Capkin M, Braddock CH, 3rd. Mediated decision support in prostate cancer screening: a randomized controlled trial of decision counseling. Patient Educ Couns. 2011;83(2):240-6. https://dx.doi.org/10.1016/j.pec.2010.06.011
39. Nadler MB, Corrado AM, Wilson BE, Desnoyers A, Amir E, Ivers N, Desveaux L. Perceived guideline clarity impacts guideline-concordant care for breast cancer screening in women age 40-49. BMC Womens Health. 2023;23(1):75. https://dx.doi.org/10.1186/s12905-023-02190-w
40. Naeim A, Sepucha K, Wenger N, et al. Participation in a personalized breast cancer screening trial does not increase anxiety at baseline. Cancer Research Conference: San Antonio Breast Cancer Symposium, SABCS. 2017;78(4 Supp 1). https://dx.doi.org/10.1158/15387445.SABCS17-PD2-14
41. Nair A, Saleem A, Selman G, et al. Feasibility of a Lung Health Clinic for early lung cancer identification in high-risk individuals in South-East London. J Thorac Oncol 2019;14(10 Supp):S521-S522. https://dx.doi.org/10.1016/j.jtho.2019.08.1088
42. Nye LE, Smith S, Knight CJ, Klemp JR. Project BRA: Breast cancer risk assessment. Journal of Clinical Oncology Conference: Annual Meeting of the American Society of Clinical Oncology, ASCO. 2022;40(16 Supp 1). https://dx.doi.org/10.1200/JCO.2022.40.16_suppl.10549
43. Padamsee TJ, Bijou C, Swinehart-Hord P, et al. Risk-management decision-making data from a community-based sample of racially diverse women at high risk of breast cancer: rationale, methods, and sample characteristics of the Daughter Sister Mother Project survey. Breast Cancer Res. 2024;26(1):8. https://dx.doi.org/10.1186/s13058-023-01753-x
44. Park S, Cochrane BB, Koh SB, Chung CW. Comparison of breast cancer risk estimations, risk perception, and screening behaviors in obese rural Korean women. Oncol Nurs Forum. 2011;38(6):E394-E401. https://dx.doi.org/10.1188/11.ONF.E394-E401
45. Piper MS, Maratt JK, Tavakkoli A, Metko V, Waljee AK, Zikmund-Fisher BJ, Saini SD. Patients are uncomfortable with the use of risk calculators to guide decisions about colorectal cancer screening: results of a large regional survey. Gastroenterology. 2017;152(5 Supp 1):S525.
46. Record SM, Thomas SM, Chanenchuk T, Baker JA, Grimm LJ, Plichta JK. Breast cancer risk assessment and screening practices reported via an online survey. Ann Surg Oncol. 2023;30(10):6219-6229. https://dx.doi.org/10.1245/s10434-023-13903-8
47. Sandiford L, D'Errico EM. Facilitating shared decision making about prostate cancer screening among African American men. Oncol Nurs Forum. 2016;43(1):86-92. https://dx.doi.org/10.1188/16.ONF.86-92
48. Saya S, McIntosh JG, Winship IM, et al. Informed choice and attitudes regarding a genomic test to predict risk of colorectal cancer in general practice. Patient Educ Couns. 2022;105(4):987-995. https://dx.doi.org/10.1016/j.pec.2021.08.008
49. Seitzman RL, Pushkin J, Berg WA. Effect of an educational intervention on women's health care provider knowledge gaps about breast cancer risk model use and high-risk screening recommendations. J Breast Imaging. 2023;5(1):30-39. https://dx.doi.org/10.1093/jbi/wbac072
50. Sen S, Ribeiro MH, Nygard M. Portinari: Communicating personalized risk in cervical cancer screening using data exploration. Cancer Epidemiology Biomarkers and Prevention Conference: AACR Conference on the Improving Cancer Risk Prediction for Prevention and Early Detection Orlando, FL United States. 2017;26(5 Suppl 1). https://dx.doi.org/10.1158/1538-7755.CARISK16-B20
51. Speare V, Dalton E, Laduca H, Grosvenor C, Kuo JZ, Lin E, Dolinsky JS. PALB2 mutation carriers: Are clinicians acting on the molecular diagnosis? Journal of Clinical Oncology Conference. 2016;34(Supp 15).
52. Tiemsani C, Boinon D, Yung MF, et al. Receipt of breast cancer risk assessment and personalized prevention information among women diagnosed with a benign breast lesion (BBL) in a one stop breast unit: a prospective assessment. Cancer Research Conference: 38th Annual CTRC AACR San Antonio Breast Cancer Symposium San Antonio, TX United States Conference Publication 2016;76(4 Supp 1). https://dx.doi.org/10.1158/1538-7445.SABCS15-P3-10-03

53. Toes-Zoutendijk E, de Jonge L, Breekveldt ECH, Korfage IJ, Usher-Smith JA, Lansdorp-Vogelaar I, Dennison RA. Personalised colorectal cancer screening strategies: Information needs of the target population. Prev Med Rep. 2023;35. https://dx.doi.org/10.1016/j.pmedr.2023.102325
54. Trevena LJ, Meiser B, Mills L, et al. Which test is best? A cluster-randomized controlled trial of a risk calculator and recommendations on colorectal cancer screening behaviour in general practice. Public Health Genomics. 2022;25(5-6):193-208. https://dx.doi.org/10.1159/000526628
55. Turhan E, Yasli G. Breast cancer risk evaluation by utilizing Gail model and association between breast cancer risk perception with early diagnosis applications among midwives and nurses working in primary health services. P R Health Sci J. 2018;37(2):98-104.
56. Turner AR. Translational genomics: The impact of Genetic Risk Score on patients and physicians. Dissertation Abstracts International: Section B: The Sciences and Engineering. 2017;77(11-B(E)).
57. Uzan C, Ndiaye-Gueye D, Nikpayam M, et al. First results of a breast cancer risk assessment and management consultation. Bulletin du Cancer. 2020;107(10):972-981. https://dx.doi.org/10.1016/j.bulcan.2020.08.003
58. Van Der Meulen MP, Korfage IJ, Van Heijningen EMB, De Koning HJ, Van Leerdam ME, Dekker E, Lansdorp-Vogelaar I. Interpretation and adherence to the updated risk-stratified guideline for colonoscopy surveillance after polypectomy - A nationwide survey. Endosc Int Open. 2020;8(10):E1405-E1413. https://dx.doi.org/10.1055/a-1190-3656
59. van Erkelens A, Sie AS, Manders P, et al. Online self-test identifies women at high familial breast cancer risk in population-based breast cancer screening without inducing anxiety or distress. Eur J Cancer. 2017;78:45-52. https://dx.doi.org/10.1016/j.ejca.2017.03.014
60. van Vugt HA, Roobol MJ, Venderbos LD, et al. Informed decision making on PSA testing for the detection of prostate cancer: an evaluation of a leaflet with risk indicator. Eur J Cancer. 2010;46(3):669-77. https://dx.doi.org/10.1016/j.ejca.2009.11.022
61. Walsh JME, Karliner L, Smith A, et al. LungCARE: encouraging shared decision-making in lung cancer screening-a randomized trial. J Gen Intern Med. 2023;38(14):3115-3122. https://dx.doi.org/10.1007/s11606-023-08189-1
62. Wegwarth O, Pashayan N, Widschwendter M, Rebitschek FG. Women's perception, attitudes, and intended behavior towards predictive epigenetic risk testing for female cancers in 5 European countries: a cross-sectional online survey. BMC Public Health. 2019;19(1):667. https://dx.doi.org/10.1186/s12889-019-6994-8
63. Wehbe A, Laws A, Katlin F, et al. Breast imaging recommendations for females <40 years of age with >=20% lifetime breast cancer risk: practice patterns at a specialized clinic. Cancer Research Conference. 2022;83(5 Supp). https://dx.doi.org/10.1158/1538-7445.SABCS22-P5-04-06
64. Wong XY, Chong KJ, van Til JA, Wee HL. A qualitative study on Singaporean women's views towards breast cancer screening and Single Nucleotide Polymorphisms (SNPs) gene testing to guide personalised screening strategies. BMC Cancer. 2017;17(1). https://dx.doi.org/10.1186/s12885-017-3781-8
65. Wong XY, Groothuis-Oudshoorn CGM, Tan CS, et al. Women's preferences, willingness-to-pay, and predicted uptake for single-nucleotide polymorphism gene testing to guide personalized breast cancer screening strategies: A discrete choice experiment. Patient Pref Adherence. 2018;12:1837-1852. https://dx.doi.org/10.2147/PPA.S171348
66. Yoon JH, Lofters A, Liu G. Feasibility pilot implementation of a low-dose CT screening program. Journal of Clinical Oncology Conference. 2018;36(7).
67. Yoon S, Goh H, Fung SM, et al. Experience and perceptions of a family health history risk assessment tool among multi-ethnic Asian breast cancer patients. J Pers Med. 2021;11(10). https://dx.doi.org/10.3390/jpm11101046
68. Yoruk S, Acikgoz A, Turkmen H, Ergor G. Risk factors and relationship between screening periodicity and risk of cervical cancer among nurses and midwives. A cross-sectional study. Sao Paulo Med J. 2019;137(2):119-125. https://dx.doi.org/10.1590/1516-3180.2018.0244230119
69. Venning B, Saya S, Lourenco RD, Street DJ, Emery JD. Preferences for a polygenic test to estimate cancer risk in a general Australian population. Genet Med. 2022;24(10):2144-54.
70. Wheeler JC, Keogh L, Sierra MA, Devereux L, Jones K, IJzerman MJ, Trainer AH. Heterogeneity in how women value risk-stratified breast screening. Genet Med. 2022;24(1):146-56.

71. Chu CD, Smith CE, Gorski J, et al. Implementation of a novel patient decision aid for women with elevated breast cancer risk who are considering MRI screening: A pilot study. Ann Surg Oncol. 2023;30(10):6152-6158.

Appendix E: Characteristics of included studies (N=63).

Source	Cancer Site	Population	Country	Data analysed	Recruitment	Risk assessment method	Measures
**Mixed Methods Studies (n=10)**							
Bancroft 2015	Prostate	Participants	United Kingdom	Pre-test and post-test survey data and interview data	Participants of a previous trial	Prostate cancer risk scores were calculated using a panel of 39 single-nucleotide polymorphisms. The risk score was provided as a percentage estimating men's risk of developing prostate cancer by the age of 80. The genetic risk score was given to men at the end of the study after the received the results of all screening procedures (PSA test, MRA, and/or prostate biopsy).	Survey:<br>1) Psychological distress (cancer worry and general & PrCa-specific screening anxiety)<br>2) Risk perception<br>Interviews:<br>3) Psychological impact of risk-based screening
Conley 2024	Breast	HCPs	USA	Cross-sectional survey data and interview data	Online (licensure databases, online search for primary care and radiology clinics in the institution's catchment area, email listservs for local professional groups); interviews conducted with subset of survey participants	No risk assessment tool used. Clinicians answered questions in response to clinical vignettes about hypothetical patients.	Survey:<br>1) Clinician and practice-level factors<br>2) Screening recommendations based on clinical vignettes<br>Interviews: Obtain an in-depth understanding of clinicians' decision-making process for breast cancer screening recommendations
DuBenske 2021	Breast	HCPs, participants	USA	Post-test survey data and analysis of audio recordings of SDM conversations between patient and physician	Primary care clinic	The BCARE-DA is an interactive, web accessible, and electronic medical record (EMR)-embedded SDM tool. Using the Breast Cancer Surveillance Consortium Risk Calculator, it determines individual baseline risk of breast cancer incidence and mortality in the next 10 years (www.healthdecision.org/tool#/tool/mammo). Physicians used the BCARE-DA during the visit and provided the risk estimate to patients.	Survey:<br>1) Decisional conflict<br>Recordings:<br>2) Evaluation of clinic visit transcripts for shared decision-making quality
Greenberg 2019	Breast	HCPs	USA	Cross-sectional survey and interview data	Community clinics	Participants used the Breast Risk Stratification Questionnaire (BRSQ), developed to identify women at potentially higher risk for breast and/or ovarian cancer due to hereditary factors.	Survey:<br>1) Clinician experiences administering BRSQ<br>2) Clinician knowledge and confidence implementing BRSQ<br>3) Clinician knowledge and confidence in cancer genetics and its utility<br>Interviews:<br>4) Clinicians' perceptions of motivators and barriers to administering BRSQ, strengths and areas for improvement from clinicians'

Source	Cancer Site	Population	Country	Data analysed	Recruitment	Risk assessment method	Measures
							conversations with clients about screening results and genetic resources.
Han 2019	Lung	Participants	USA	Pre-test and post-test survey data and interview data	Lung cancer screening program at tertiary care hospital	A simple user interface was developed to display model estimates using the PLCOm2012 risk prediction model textually and visually. Clinicians enter the patient's risk factor information and communicate the patient's risk estimate using standardized language.	Survey:<br>1) Perceived lung cancer risk<br>2) Perceived uncertainty of lung cancer risk<br>3) Personal risk thresholds for wanting screening<br>4) Interest in lung cancer screening<br>Interviews:<br>5) Perceptions of the value of personalized cancer risk information and its impact of their screening decisions
Liow 2022	Breast	Participants	Singapore	Focus group data and cross-sectional survey data	For focus groups, flyers were sent out to non-profit organizations via social media and email. For the survey, data was collected as part of a larger pilot study implementation risk-based breast cancer screening.	Survey participants were patients of a larger pilot study (BREATHE) which uses the Gail model and breast cancer polygenic risk to determine individual breast cancer risk. At the time this data was collected, participants anticipated receiving their risk score in about 3 months.	Focus groups:<br>1) Perceived barriers to mammography attendance and adherence, motivations for attending mammography, views towards personalized risk-based reports and genetic testing.<br>Surveys:<br>2) Emotion-related responses in anticipation of receiving their risk profile.
Lowery 2022	Lung	HCPs	USA	Interview data	Veterans Affairs medical centers	The DecisionPrecision tool, a provider-facing web-based decision support tool for lung cancer screening decisions, was used in a clinic by the provider to tailor the LCS discussion to a patient's individualized lung cancer risk and estimated net benefit.	Implementation strategy, usability of the tool, and usefulness of the tool for LCS discussions.
Puzhko 2019	Breast	HCPs	Canada	Small group deliberations data (survey data was to assess quality of the sessions and are not relevant to this review)	Primary care clinic; Community health centers	Participants were given information about the Breast and Ovarian Analysis of Disease Incidence and Carrier Estimation Algorithm (BOADICEA) risk prediction model, used to calculate 10-year and lifetime age-specific risks of breast cancer and probabilities to be a mutation carrier, but did not use the model.	Small group deliberations were conducted to identify implementation barriers and optimization strategies for risk-stratified breast cancer screening.
Rainey 2018	Breast	HCPs	Netherlands, United Kingdom, Sweden	Qualitative (participant statements) and quantitative data (statement rating scores	Online (research team professional networks)	No risk assessment tool used.	Women's perceptions related to personalized risk-based breast cancer screening and prevention, such as barriers, worries, fears, and advantages were collected as statements and organized in clustering maps to identify salient themes.

Source	Cancer Site	Population	Country	Data analysed	Recruitment	Risk assessment method	Measures
				and correlations)			
Schroy 2015	Colorectal	HCPs	USA	Interview data and cross-sectional survey data	Academic medical center	No risk assessment tool used.	Interviews:<br>1) Factors influencing CRC screening recommendations for average-risk patients, relative importance of risk stratification in decision making, receptivity to using a risk assessment tool, and features to enhance utilization.<br>Survey:<br>2) Willingness to incorporate patient-oriented decision aid<br>2) Frequency of using electronic provider-oriented risk stratification tool, features that facilitate use, types of patients who might be candidates for risk assessment, formatting preferences, how to label an individual based on risk.
**Qualitative Studies (n=22)**							
Bas 2023	Cervical	Participants	Netherlands	Focus group data	General medical practices	No risk assessment tool used. Focus groups started with a short presentation about the current Dutch cervical cancer screening program including the triage of hrHPV-positive women.	Focus groups explored attitudes to implementing a risk-based triage strategy in the cervical cancer screening program in the Netherlands.
Blouin-Bougie 2021	Breast	HCPs	Canada	Interview data	Primary care clinic; Hospital; Community clinic	No risk assessment tool used.	Interviews sought to understand the intricacies of breast cancer risk stratification from the perspective of those directly involved in its implementation and execution.
Dodd 2024	Lung	HCPs	Australia	Focus group data	Online (from Primary Health Networks across New South Wales, contacts of research team, snowball sampling)	No risk assessment tool used. The researchers developed a structured presentation about LCS, including an overview of LCS trials and the proposed risk assessment tool (PLCOm2012).	Focus groups explored the implementation potential of lung cancer screening in Australia.
French 2022	Breast	HCPs	United Kingdom	Focus group data	Breast cancer screening service centers part of the NHS	No risk assessment tool used.	Focus groups identified important modifications to the current breast cancer screening pathway and to prioritize suggestions from different professional groups.
Furst 2018	Breast	HCPs	Germany	Focus group data	Gynecology department of a university	No risk assessment tool used.	Focus groups gained insights into current counselling practices, counselling for individualized mammography screening, and implementation of individualized screening.
Hawkins 2022	Breast	HCPs	United Kingdom	Interview data	Participants of a previous trial (HCPs from the BC-Predict	No risk assessment tool used. The BC-Predict study had breast cancer risk	Interviews explored experiences of running BC-Predict and view on implementing risk-stratified screening nationally.

Source	Cancer Site	Population	Country	Data analysed	Recruitment	Risk assessment method	Measures
					study who were part of 3 NHS Breast Screening Program sites)	assessment using the Tyrer-Cuzick risk model.	
He 2018	Breast	Participants	USA	Focus group data	In-person, with participants recruited through multiple settings (e.g. supermarkets, social media, local newspaper ads)	No risk assessment tool used. The discussions were centred around an information sheet which included a proposed risk-based screening approach.	Focus groups explored women's prior mammography experiences, knowledge of breast cancer risk factors, concepts of breast cancer risk, and reactions to an information sheet about risk-based mammography recommendation.
Hindmarch 2023	Breast	Participants	United Kingdom	Interview and focus group data	Online and in-person (advertisements on social media platforms, noticeboards in public buildings, community organizations)	No risk assessment tool used. Participants were presented with hypothetical scenarios about self-reported assessment of breast cancer risk factors, a saliva sample for assessment of polygenic risk and mutations in high-risk genes and a low-dose mammogram for assessment of breast density.	Interviews and focus groups assessed women's views and feelings on introducing breast cancer risk assessment, preferences for access to and delivery of a breast cancer risk assessment service, and their information and support needs.
Kelley-Jones 2021	Breast	Participants	United Kingdom	Interview data	Survey panel (online)	No risk assessment tool used. The concept of risk-stratified breast screening was introduced and participants reacted to screening scenarios based on different risk levels.	Interviews explored attitudes towards breast cancer, understanding of NHS breast screening programs, and perceived risk, cognitive and emotional responses to personal risk assessment (PRA), willingness to undergo various tests and provide personal health information, and reactions to screening scenarios based on different risk levels.
Laza-Vasquez 2022a	Breast	HCPs	Spain	Focus group data	Primary care clinic; Hospital	No risk assessment tool used. Moderators started by presenting a proof-of-concept study about risk-based screening.	Focus groups assessed the feasibility and acceptability of risk-based screening among health professionals.
Lippey 2019	Breast	Participants	Australia	Focus group data	Participants of a previous trial	No risk assessment tool used. During the focus group, there was a 10 minute presentation about risk-based screening and a potential program protocol with variation of frequency and modality of screening for different risk groups.	Focus groups explored breast cancer screening experiences, risk perceptions, and perceptions of a potential program of individualized screening with possible screening frequency and modality variations for different risk groups.
Matthias 2020	Colorectal	HCPs, Participants	USA	Interview data	Veteran Affairs medical centers; academic medical center	The tool was a five-variable risk prediction tool for advanced colorectal neoplasia that uses variables (age, sex, CRC in a first-degree relative, cigarette smoking, and waist circumference) to estimate the risk of advanced neoplasia. The tool generates a score and has four risk strata ranging from very low to high risk. HCPs and participants were shown the tool but did not use it.	Individual interviews were conducted to understand experiences related to colorectal cancer (CRC) screening and a new risk prediction tool. The questions focused on CRC screening experiences and impressions of the new risk prediction tool.
McWilliams 2020	Breast	HCPs	United Kingdom	Interview data	Online (membership/guideline	No risk assessment tool used.	Interviews assessed stratification of breast cancer screening for low-risk women,

Source	Cancer Site	Population	Country	Data analysed	Recruitment	Risk assessment method	Measures
					author lists relevant to national cancer screening programs)		focusing on feasibility, implementation, low-risk threshold, screening interval length, information provision, informed decision-making, and potential implications (from service, policy and public perspectives).
McWilliams 2021	Breast	Participants	United Kingdom	Interview data	Participants of a previous trial	The Tyrer-Cuzick model was used to provide the 10-year risk of breast cancer in four risk categories: below average (or low), average, above average (or moderate) and high. Participants received their risk score as part of the larger BC-Predict study.	Interviews focused on participants' experiences in BC-Predict, their perceptions of receiving a low-risk estimate, and views on reducing screening frequency for low-risk women.
McWilliams 2023	Breast	Participants	United Kingdom	Interview data	Participants of a previous trial	Tyrer-Cuzick model. Women in the BC-Predict study received their risk feedback via letter about 6-8 weeks after their negative mammogram results.	This study explored women's experience of participating in BC-Predict, thoughts and feelings about their personal risk and any subsequent behavior change.
Rainey 2019	Breast	Participants	Netherlands, United Kingdom, Sweden	Focus group data	Participants of a previous trial	The Tyrer-Cuzick model was used to provide only UK women with a breast cancer risk score. Women in Netherlands and Sweden were not given their personal breast cancer risk.	Participants (Netherlands and Sweden) were shown three vignettes describing hypothetical women at different levels of risk and their recommended tailored screening and prevention pathway. Participants from the United Kingdom had previously received their risk score and were not shown vignettes. Focus groups explored women’s thoughts and feelings regarding having their breast cancer risk assessed, and subsequent screening and preventative options.
Rainey 2020a	Breast	Participants	Netherlands, United Kingdom, Sweden	Focus group data	Participants of a previous trial	The Tyrer-Cuzick model was used to provide only UK women with a breast cancer risk score. Women in Netherlands and Sweden were not given their personal breast cancer risk.	Focus groups were conducted to explore women's perceptions of the organization of risk-based breast cancer screening and prevention. Participants from the Netherlands and Sweden discussed hypothetical organizational scenarios and British participants discussed their actual experiences.
Roberts 2021	Lung	Participants	USA	Interview data	Veteran Affairs medical centers	Risk-Based NLST Outcomes Tool (RNOT), an online tool that calculates risk of lung cancer diagnosis and death with and without lung cancer screening, and false-positive risk estimates. Participants used the RNOT during the interview and received their risk score.	Interviews assessed participants' perceptions of lung cancer risks, screening outcomes, and the utility of the Risk of Overdiagnosis Net (RNOT) tool.
Sierra 2021	Breast	Participants	Australia	Interview and focus group data	Screening cohort; clinic-based familial cancer service	No risk assessment tool used. Discussions included a presentation on genomic-based risk.	Focus groups and interviews were conducted to explore women's perceptions of polygenic breast cancer risk assessments (PBCRAs).
Walker 2017	Colorectal	HCPs	Australia	Interview data	Primary care clinic	The Colorectal cancer RISk Prediction tool (CRISP) uses the 'Freedman' CRC risk model and was designed to assist clinicians in	Interviews explored HCPs' acceptability of using CRISP, its usability, and implementation strategies.

Source	Cancer Site	Population	Country	Data analysed	Recruitment	Risk assessment method	Measures
						collecting patient risk information and providing screening recommendations.	
Woof 2020	Breast	Participants	United Kingdom	Interview data	Community outreach events	No risk assessment tool used. Interviews used the BC-Predict study procedure to explain to women how a risk estimate is obtained.	Interviews focused on participants' experiences with breast cancer screening and their opinions on a proposed new service involving online risk questionnaires and mammograms for risk calculation.
Woof 2021	Breast	HCPs	United Kingdom	Interview and focus group data	Primary care clinics, NHS Breast Screening Program	No risk assessment tool used.	Interviews and focus groups evaluated the implementation and organization of a low-risk pathway in breast cancer screening, and its potential impact on HCPs, the general population, and public opinion.
**Quantitative Studies (n=31)**							
Braithwaite 2023	Breast	HCPs, Participants	USA	Cross-sectional survey data	Clinicians recruited from primary care Practice-Based Research Network (PBRN); Patients recruited from primary care clinics	Participants received a tailored infographic booklets based on their lifetime breast cancer risk, calculated using NCI's Breast Cancer Risk Assessment Tool (BCRAT). The Lee-Schonberg Index within the ePrognosis breast cancer screening module was used to calculate screening benefits and harms.	Clinicians:<br>1) Perspectives on screening mammography in patients age 75 and older<br><br>Patients:<br>1) Intervention acceptability<br>2) Overall satisfaction with materials<br>3) 5-year and lifetime perceived breast cancer risk (pre and post)<br>4) Cancer worry oriented to the decision to continue or discontinue mammography<br>5) Intentions to continue screening mammography
French 2018	Breast	Participants	United Kingdom	Randomized controlled trial data (qualitative results are not analysed as they were for stimuli development)	Participants of a previous trial	Two types of risk assessment used (i.e. Tyrer-Cuzick model vs Tyrer-Cuzick model + single-nucleotide polymorphisms [SNPs]). Women received risk assessments as part of the PROCAS study and received posted letters with their risk estimates and explanatory leaflets.	1) State anxiety<br>2) Breast cancer worry<br>3) Perceived relative risk of developing breast cancer<br>4) Intentions to change for five health-related behaviours related to breast cancer prevention<br>Participants in the T-C, or T-C(+SNPs) groups but not the comparison group were also asked to complete the following measures:<br>1) Satisfaction with the information<br>2) Understanding of test result
Ghanouni 2020a	Breast	Participants	United Kingdom	Cross-sectional survey data	Survey panel, survey administered in-person in participants' homes	No risk assessment tool used. Women received summary information about breast cancer, screening, risk assessment, and risk stratification.	1) Perceived susceptibility to breast cancer, how often they worried about getting breast cancer<br>2) Mammography history<br>3) Perceptions of using personal information to determine risk<br>4) Willingness to have breast cancer risk estimated and reasons why<br>5) Thoughts of tailoring screening based on

Source	Cancer Site	Population	Country	Data analysed	Recruitment	Risk assessment method	Measures
							risk, willingness to have screening invitations more often than 3 years if very high risk, or less often than 3 years if very low risk.
Ghanouni 2020b	Breast	Participants	United Kingdom	Post-test survey data	Online	No risk assessment tool used. Participants read a vignette outlining two screening strategies, followed by randomly ordered information on screening benefits and risks.	1) Screening preference<br>2) Comprehension of information<br>3) Perceived benefits and risks of screening
Koitsalu 2016	Breast, prostate	Participants	Sweden	Cross-sectional survey data	Online (Swedish population registry)	No risk assessment tool used.	1) Interest in cancer risk and underlying reasons (intention to know their cancer risk, underlying reasons for wanting to know their risk, underlying reasons for not wanting to know)<br>2) Acceptability of screening tailored to risk<br>3) Acceptability of the communication structures (conveying personal and genetic information and how cancer risk should be conveyed)
Koitsalu 2021	Prostate	Participants	Sweden	Pre-test and post-test survey data	Participants of a previous trial	Participants' prostate cancer risk were calculated using the STHLM3 model (uses a combination of plasma protein biomarkers, genetic polymorphisms, and clinical variables). Participants received a response letter with their test results and informing them as to which PCa risk level they belonged to and tailored recommendations.	1) PCa worry and perceived vulnerability<br>2) PCa knowledge<br>3) Attitude toward PCa testing and health behaviour
Lapointe 2022	Breast	HCPs	Canada	Cross-sectional survey data	Online (professional associations, health care institution newsletters and communication platforms, research team networks)	No risk assessment tool used.	1) Familiarity with polygenic risk score, level of knowledge<br>2) Status of their training and future professional curriculum<br>3) Preferences for continuing professional education
Lapointe 2023	Breast	HCPs	Canada	Cross-sectional survey data	Online (research team professional networks)	No risk assessment tool used.	1) Attitudes toward breast cancer screening recommendations in a context of risk stratification<br>2) Attitudes toward their role and scope of practice within a risk-stratified breast cancer screening approach<br>3) Views toward the necessary enhancements to the healthcare system required<br>4) Views toward the professional group that should play a role
Lau 2015	Lung	Participants	USA	Pre-test and post-test survey data	Online (web-based)	Participants used the ShouldIScreen.com lung cancer screening decision aid which includes the PLCOm2012 risk prediction model. Participants received their risk	1) Decisional conflict<br>2) Concordance (match between eligibility and preference for screening)

Source	Cancer Site	Population	Country	Data analysed	Recruitment	Risk assessment method	Measures
						assessment while using the web-based decision aid.	3) Acceptability of decision aid<br>4) Knowledge of LCS benefits and harms
Laza-Vasquez 2022b	Breast	HCPs	Spain	Cross-sectional survey data	Online (members of healthcare-related societies and scientific groups)	No risk assessment tool used.	1) Advantages and disadvantages of risk-based screening for the health of women with an individual risk of breast cancer higher or lower than the population average<br>2) Advantages of risk-based screening, in relation to current screening, for the Spanish National Health System<br>3) Barriers and facilitators for implementation considering the advantages and disadvantages
Lipkus 2018	Colorectal	Participants	USA	Post-test survey data	Survey panel (online)	Participants used the Your Disease Risk (see http://www.yourdiseaserisk.wustl.edu) tool which assesses CRC risk (see Schroy 2011 for a detailed description of YDR). Participants received their risk score in the form of a comparative risk estimate.	1) Perceived accuracy of risk estimate<br>2) Emotional reactions to risk estimate<br>3) Perceived usefulness of risk estimate<br>4) Risk appraisal<br>5) Screening intention
Lippey 2023	Breast	Participants	Australia	Pre-test and post-test survey data	Women participating in the BreastScreen Victoria (BSV) program	No risk assessment tool used. Participants used DEFINE, a decision aid to support the process of choosing between current or risk-stratified screening. DEFINE includes links to iPrevent and Tyrer-Cuzick risk calculators.	1) Risk perception of developing breast cancer<br>2) Breast cancer worry and anxiety<br>3) Understanding of risk-stratified screening<br>4) Interest in risk-stratified screening overall<br>5) Acceptability of varying screening levels<br>6) Acceptability of genetic testing incorporated into screening and risk assessment<br>7) Informed decision making<br>8) Satisfaction with decision
Loft 2024	Breast	Participants	Denmark	Cross-sectional survey data	Survey panel (online)	No risk assessment tool used.	1) Opinions on personalized breast cancer screening frequency based on individual risk.<br>2) Attitudes towards knowing one's risk of developing breast cancer.<br>3) Comfort level with genetic testing through blood samples for breast cancer risk estimation.<br>4) Preferences for mammography frequency if identified as high-risk for breast cancer.<br>5) Preferences for mammography frequency if identified as low-risk for breast cancer.
Mbuya-Bienge 2021	Breast	Participants	Canada	Cross-sectional survey data	Survey panel (online)	No risk assessment tool used.	1) Attitudes towards breast cancer risk assessment and risk-based screening<br>2) Being comfortable in providing information for breast cancer risk assessment<br>3) Willingness to have breast cancer risk assessed and tailored screening frequency

Source	Cancer Site	Population	Country	Data analysed	Recruitment	Risk assessment method	Measures
Meisel 2015	Breast	Participants	United Kingdom	Cross-sectional survey data	Population-based 'Opinions and Lifestyle' survey administered by the UK Office of National Statistics	No risk assessment tool used. The survey module included a brief explanation of genes, DNA, and genetic testing.	1) Attitudes to risk-stratified breast cancer screening<br>2) Personal acceptability of modified breast screening frequency<br>3) Attitudes towards genetic testing generally were assessed to be sure that acceptability of stratified screening was not influenced by views about genetic testing.
Miller 2021	Colorectal	Participants	USA	Pre-test and post-test survey data (sub-analyses of RCT results where intervention groups were combined for before-and-after analysis)	Online (web-based)	No risk assessment tool used. Half the participants received personalized risk management messages based on their self-reported risk factors, but did not receive risk scores.	1) Lifetime risk accuracy<br>2) Behavioural intentions related to CRC screening, diet, and physical activity
Morman 2017	Breast	Participants	USA	Cross-sectional survey data	Women who had underwent a screening mammogram at two imaging centers	One or more of three risk models was used as appropriate for each woman (Gail, Claus, and Tyrer-Cuzick). Participants received their risk assessment in a mailed letter disclosing the results of the breast cancer risk assessment (BCRA) and recommendations.	1) Women's perceived risk of breast cancer<br>2) Impact of recommendation for genetic testing<br>3) Compliance with the breast health care and genetic counselling recommendations<br>4) Discussing the results of the BCRA with a physician<br>5) Value of BCRA to women
Piper 2018	Colorectal	Participants	USA	Cross-sectional survey data	Veteran Affairs medical centers	No risk assessment tool used. The survey presented a detailed hypothetical scenario about CRC screening initiation and cessation.	1) Attitudes toward use of age in the decision to start and stop CRC screening<br>2) Attitudes toward life expectancy and CRC risk calculators<br>3) Attitudes toward stopping low-value CRC screening<br>4) Factors associated with comfort with low-value screening cessation.
Pruitt 2024	Breast	HCPs	USA	Cross-sectional survey data	Mailed surveys to primary care physicians in two areas of Texas	No risk assessment tool used. Physicians were asked about use of the Gail model.	1) Whether the physician used the Gail model<br>2) Application of the Gail score<br>3) Use of chemoprevention
Rainey 2020b	Breast	Participants	Netherlands	Post-test survey data	Participants of a previous trial	No risk assessment tool used. Participants were randomly assigned to read one of four hypothetical breast cancer risk scenarios (i.e., low, average, moderate, or high) with tailored screening intervals and prevention advice.	1) Perceived need for supplemental screening outside of the national screening program<br>2) Interest in knowing breast cancer risk<br>3) Perceived need for breast self-examination based on personalized screening interval

Source	Cancer Site	Population	Country	Data analysed	Recruitment	Risk assessment method	Measures
Rainey 2022	Breast	Participants	United Kingdom	Cross-sectional survey data	Participants of a previous trial	Participants were previously given their breast cancer risk, calculated using the Tyrer-Cuzick model, as part of the PROCAS study (i.e. low, average, moderate, high)	Early detection behaviours included: 1) intent to request supplemental mammography outside the national screening program, 2) increased breast self-examination.<br>Preventive behaviours included: 3) started with preventative medication, 4) changed diet, 5) changed physical activity levels, 6) changed alcohol intake.
Resong 2024	Lung	HCPs	USA	Survey data	Online (providers on Texas-based providers registry and physicians, physician assistants, and nurse practitioners at the 2022 Texas Academy of Family Physicians Primary Care Summit)	No risk assessment tool used.	1) Current practices in PCP's practices<br>2) Thoughts about 2021 USPSTF lung cancer recommendations<br>3) Opinions on personalized lung cancer screening (use if risk calculator and biomarkers)
Rupert 2013	Breast	HCPs, Participants	USA	Pre-test and post-test survey data	Primary care clinic	Cancer in the Family, an online clinical decision support tool, calculated women's hereditary breast and ovarian cancer (HBOC) risk and promoted shared patient-provider decisions about screening. Participants received their BRCA mutation risk as part of using the tool prior to their wellness exam.	1) Tool use and family history collection<br>2) HBOC and BRCA knowledge<br>3) Risk results and perceptions<br>4) Patient-provider discussions and decisions.
Schroy 2011	Colorectal	Participants	USA	Pre-test and post-test survey data	Primary care clinic	Half of the participants used a CRC screening decision aid plus a CRC risk assessment tool ("Your Disease Risk" [YDR]; http://www.yourdiseaserisk.wustl.edu). YDR assesses CRC risk with questions on family history, BMI, CRC screening (reassessment), history of cancers, aspirin use, inflammatory bowel disease, use of multivitamin, calcium and vitamin D supplements, consumption of milk products, meat intake, alcohol consumption, physical activity, and estrogen replacement. The other half of the participants used the CRC screening decision aid only.	1) Patient knowledge<br>2) Patients preferences<br>3) Satisfaction with the decision-making process<br>4) Screening intentions<br>5) Test concordance (agreement between patient preference and test ordered)
Schroy 2016	Colorectal	HCPs, Participants	USA	Pre-test and post-test survey data	Academic medical center	The Advanced Colorectal Neoplasia Index (ACNI) consists of 6 independent predictors of risk (age, sex, race/ethnicity, smoking history, daily alcohol intake, and use of nonsteroidal anti-inflammatory drugs [NSAIDs]). Risk scores were provided to those that were randomized to the group that completed risk assessment.	1) Concordance between patient preference and test ordered<br>2) Patient satisfaction with the decision-making process<br>3) Screening intentions<br>4) Test completion rates<br>5) Provider satisfaction

Source	Cancer Site	Population	Country	Data analysed	Recruitment	Risk assessment method	Measures
Segar 2024	Breast	Participants	Malaysia	Cross-sectional survey data	Online (research team's social networks, social media platforms)	No risk assessment tool used.	1) General breast cancer awareness (risk factors, signs and symptoms, benefits of regular breast cancer screening, potential consequences of screening, source of information)<br>2) Knowledge of personalized risk-stratified breast cancer screening<br>3) Attitudes towards personalized risk-stratified breast cancer screening.
Turner 2016	Prostate	Participants	USA	Randomized controlled trial data	Primary care clinic	Participants received a report of lifetime risk of PCa based on either: family history only, or family history plus genetic risk score (using single-nucleotide polymorphisms (SNPs) from saliva samples). Participants met with a genetic counsellor to receive their personal risk report.	1) State and trait anxiety<br>2) Risk recall (open-ended question of the risk given to them)<br>3) Discussion with doctor about having a PSA test<br>4) PSA testing at 3 months (primary outcome)
Veron 2018	Breast	Participants	France	Post-test survey data	Community clinic	Mammorisk. Participants received their risk score, but no information on how it was delivered (study is published as an abstract).	1) Percentage of women accepting to participate in receiving BC risk<br>2) Women's awareness, anxiety, and satisfaction after using the tool
Veron 2023	Breast	Participants	France	Pre-test and post-test survey data	Community practices	Participants were those who were at increased non-genetic risk of breast cancer using the CANRISK-based 5-year risk of invasive breast cancer > 2.5%	1) Perception of knowledge on risk<br>2) Perception of prevention and screening<br>3) Self-evaluated lifetime probability and compared probability of breast cancer
Weigert 2018	Breast	HCPs	USA	Cross-sectional survey data	Hospital	The MammoRisk software was used with women undergoing routine mammography examinations.	Ratings of the software's interface, the patient questionnaire, the amount of time needed to fill in the patient information, and the mammographer's readiness to integrate the software tool into daily practice.
Wu 2013	Breast, colorectal	HCPs, Participants	USA	Pre-test and post-test survey data (patients and providers)	Primary care clinic	MeTree is a patient-facing familial health history (FHH) collection tool with clinical decision support (CDS) and uses a Gail score. MeTree© then risk-stratifies patients for five diseases (breast, ovarian and colorectal cancer, thrombosis, and hereditary cancer syndromes) and recommends risk-guided prevention strategies endorsed by evidence-based guidelines. Participants received their risk score while using MeTree at a clinic kiosk.	1) Ease of use of MeTree<br>2) Satisfaction with MeTree<br>2) Impressions of MeTree at 3 months

Appendix F: Characteristics of participants.

Table F1: Characteristics of general public participants (N=25,638, 42 studies[a]).

Source	Sample Size	Sex/Gender[b]	Age (Years)	Race and Ethnicity	Education Level	Income Level or SES	Insurance Status
Bancroft 2015	95 (95 completed surveys, of which 26 participated in interviews)	Sex: 100% male	Range = 40-69 years old.	NR	NR	NR	NR
Bas 2023	28	Sex: 100% female	Mean (SD) = 45.2 (7.6)	NR	50% lower-intermediate education, 50% higher education	NR	NR
Braithwaite 2023	14	Sex: 100% female	Mean (SD) = 79.1 years (3.0)	57.1% White, 42.9% Black	57.1% high school graduate or less, 21.4% some college, 21.4% completed college or higher	35.7% $10,000-40,000, 14.2% $40,001-80,000, 28.6% >$80,000, 21.4% prefer not to answer	NR
DuBenske 2021	53	Sex: 100% female	Included range = 40-49 years	94.4% White	72.2% college- educated	52.8% with annual household income greater than US$100,000	NR
French 2018	765	Sex: 100% female	Mean (SD) = 56.2 (4.67)	92.2% White, 1% Black, 1.1% Asian, 5.7% other	NR	NR	NR
Ghanouni 2020a	933	Sex: 100% female	Mean (SD) = 54.9 (9.3)	82.2% White, 16.9% Other	30.8% Graduate level and above, 66.9% Other qualifications	51.8% working	NR
Ghanouni 2020b	698	Sex: 100% female	Mean (SD) = 53.4 (7.9)	NR	97% spoke English as first language	NR	NA (universal healthcare)
Han 2019	60 (60 completed surveys, of which 17 participated in interviews)	Sex: Surveys = 41% female; Interviews = 35% female	Surveys: Mean (SD) = 63.2 (5.2) Interviews: Mean (SD) = 64.9 (4.0)	NR	NR	NR	NR
He 2018	29	Sex: 100% female	23 (79.3%) 50-74 years old, 6 (20.7%) under 49 years old	100% White	82.3% college grad or higher, 6.9% some college, 10.3% high school graduate	62.1% working, 27.6% retired, 10.3% unemployed	24.1% Medicare or Medicaid, 75.9% other insurance
Hindmarch 2023	37	Sex: 100% female	49% age 30-33, 19% age 34-36, 32% age 37-39	78% White British, 10% Black African, 3% Indian, 3% White (other), 3% mixed (White/Arab), 3% mixed (English Caribbean)	NR	41% lived in an area with high deprivation decile, 49% medium deprivation decile, 10% low deprivation decile	NR
Kelley-Jones 2021	25	Sex: 100% female	7 40-49; 9 50-59; 9 60-70 (range 40-68)	76% White, 8% Black, 16% Asian	1 no qualifications, 4 O Level, 6 A Level, 6	13 managerial/professional	NR

					university degree, 5 Masters or higher, 3 other	occupational grade, 12 manual/semi-skilled	
Koitsalu 2016	2822	Sex: 48.1% female	Men: 25% age 20-39, 41% age 40-59, 33% age 60-74<br>Women: 29% age 20-39, 39% age 40-59, 31% age 60-74	NR	Men: 52% low education, 42% high education<br>Women: 43% low education, 50% high education	Men: 30% low income, 67% high income<br>Women: 46% low income, 50% high income	NR
Koitsalu 2021	994	Sex: 100% male	17.3% age 50-54; 21.9% age 55-59; 24.4% age 60-64; 33.0% age 65-69; 3.1% age 70 and above	NR	8.1% elementary school, 28.7% upper secondary school, 39.7% university, 16.2% other	NR	NR
Lau 2015	60	Sex: 50% female	Mean (SD) = 60.6 (7.3)	88% White, 12% Black	2% less than high school, 7% high school graduate, 7% some training after high school, 22% some college, 28% college graduate, 35% postgrad or professional degree	NR	NR
Liow 2022	1047 (54 from focus groups, 993 from surveys)	Sex: 100% female	Focus groups: Median age = 37.5 years (range 13-31)<br>Surveys: Median age = 49 years (IQR 43-54)	Focus group: 86.7% Chinese, 3.7% Indian, 1.8% Malay, 7.5% others; Survey: 77% Chinese, 6% Indian, 12% Malay, 5% others	NR	NR	NR
Lipkus 2018	560	Gender: 53.2% female	Mean (SD) = 57.9 (6.2)	76.8% White, 7.3% Black, 3.4% other, 9.5% Hispanic	9.8% less than high school, 36.2% had high school education or less, 28.4% some college education, 25.5% college graduates	46.9% working full time, 12.2% work part time, 13.2% unemployed, 27.7% retired	85.4% have health insurance
Lippey 2019	52	Sex: 100% female	Mean (SD) = 61 (5.18), range 48-72	NR	5.8% did not complete high school; 13.5% high school; 42.3% trade/apprentice/certificate/diploma; 38.5% university education.	Using the postcode IRSAD measures of socioeconomic disadvantage on a scale 1 (least advantaged) to 5 (most advantaged): IRSAD 1 (0%), IRSAD 2 (3.8%), IRSAD 3 (17.3%), IRSAD 4 (52.8%), IRSAD 5 (19.2%), missing (5.8%)	NR
Lippey 2023	242	Sex: 100% female	Mean = 66, range 40-74	NR	67% University degree, 19% High school, 10% Did not complete high school, 1% Trade, 3% Missing	10% Lowest quintile, 17% Second quintile, 19% Third quintile, 22% Fourth quintile, 32% Highest quintile	NR

Loft 2024	5001	Sex: 100% female	Mean = 59.5	NR	7.0% less than 10 years of education, 45.8% 10-12 years of education, 46.7% more than 12 years of education	NR	NR
Matthias 2020	15	Sex: 47% female	Mean (SD) = 59.8 (7.4)	40% White, 60% Black	NR	NR	NR
Mbuya-Bienge 2021	4219	Sex: 100% female	24.8% age 30-39; 24.8% age 40-49; 25.3% age 50-59; 25.1% age 60-69	80.8% White, 16.1% other	27.7% high school diploma or less; 43.4% non-university certificate or post-secondary diploma; 28.9% university diploma.	7.7% Less than $20,000; 14.7% $20,000-$39,999; 17.3% $40,000-$59,999; 13.9% $60,000-$79,999; 33.1% $80,000 or more; 13.3% Don't know/Prefer not to answer.	NR
McWilliams 2021	23	Sex: 100% female	46-54 years = 16 (69.6%)<br>55-64 years = 3 (13%)<br>65-74 years = 4 (17.4%)	87.0% White, 4.3% Black, 4.3% Asian, 4.3% other	8.7% A level or equivalent; 13.0% Diploma; 43.5% Degree; 8.7% Postgraduate certificate/diploma; 21.7% Postgraduate degree.	Using a deprivation index from 1 (most deprived) to 10 (least deprived): 1 = 4.3%; 2 = 8.7%; 3 = 17.3%; 4 = 4.3%; 5 = 30.4%; 6 = 0%; 7 = 0%; 8 = 26.1%; 9 = 4.3%; 10 = 4.3%.	NR
McWilliams 2023	40	Sex: 100% female	32.5% age 47-54, 65% age 55-64, 2.5% age 65-74	90% White British or Irish, 2.5% Black African or Caribbean, 2.5% other, 5% unknown	NR	7.5% lived in an area with high deprivation decile, 30% in medium deprivation decile area, 62.5% in low deprivation decile area	NR
Meisel 2015	942	Sex: 100% female	Mean (SD) = 47 years (15.6), range 18-74 years.	92.8% White, 7.2% other	26.8% University educated and above; 73.2% Below university degree.	NR	NR
Miller 2021	419	Gender: 67% female	Mean (SD) = 58.5 (6.3)	33.4% White, 33.4% Black, 33.2% Hispanic	NR	41.3% employed part-time or full-time; 43.0% had annual household income of $50,000 or higher	NR
Morman 2017	69	Sex: 100% female	Mean (SD) = 50.4 (9.1)	81.2% White, 15.9% Black, 2.9% other	NR	NR	82.6% private insurance; 14.5% Medicare/Medicaid; 2.9% uninsured
Piper 2018	1054	Sex: 5.8% female	0.1% age 40- 49; 26.3% age 50-59; 56.8% age 60-69; 13.6% age 70-79; 3.2% age 80-89	85.9% White, 9.5% Black, 1.9% other, 2.7% Hispanic	49.7% completed education beyond high school; 5.2% had either not graduated high school or earned a vocational or technical degree.	NR	NR
Rainey 2019	143	Sex: 100% female	Median age: Sweden = 67.0, Netherlands = 57.5, UK = 56.0	NR	Median years of education: UK = 15.0,	NR	NR

					Netherlands = 17.0, Sweden = 21.0.		
Rainey 2020a	143	Sex: 100% female	Median age: Sweden = 67.0, Netherlands = 57.5, UK = 56.0	NR	Median years of education: UK = 15.0, Netherlands = 17.0, Sweden = 21.0.	NA	NA
Rainey 2020b	942	Sex: 100% female	Mean (SD) = 59.0 (6.3)	NR	26.4% lower education; 39.5% higher secondary education; 34.1% higher vocational qualification.	NR	NR
Rainey 2022	325	Sex: 100% female	Mean (SD) = 61.3 (4.9)	NR	20.9% lower education; 30.2% higher secondary education; 40.3% higher vocational qualification.	NA	NA
Roberts 2021	10	Sex: 40% female	60 years old or less = 20%; Above 60 years = 80%	NR	60% less than college; 40% college education.	NR	NR
Rupert 2013	48	Sex: 100% female	20.8% age 21-40; 43.8% age 41-50; 35.4% age 51-60.	89.5% White, 2.1% Black, 2.1% Native Hawaiian, 4.2% Hispanic, 2.1% multiple races.	High school or less = 6.3%; some college or technical school = 33.3%; college degree (4-year) = 41.7%; graduate degree = 18.8%	Less than $40,000 = 4.2%; $40,000-79,999 = 10.4%; $80,000-99,999 = 16.7%; $100,000 or more = 66.7%	NR
Schroy 2016	341	Sex: 52.8% female	Mean (SD) = 56.4 (6.3)	22% White, 61% Black, 17% other, 6.7% Hispanic	62.2% high school or less; 37.8% greater than high school.	NR	27.3% private/HMO; 54.8% Medicare/Medicaid; 12.0% free care; 5.0% other; 1.2% none.
Schroy 2011	666	Sex: 59.6% female	83.2% under 65 years; 16.8% 65 and above	62.8% Black, 33.8% White, 1.4% Asian, 2.1% other, 5.7% Hispanic	74.2% high school and above, 24.5% less than high school	NR	34.2% private/HMO; 49.4% Medicare/Medicaid; 5.9% free care; 1.7% none
Segar 2024	201	Sex: 100% female	74.6% under 39 years, 25.4% 40 years and above	18.9% Malay, 35.3% Chinese, 19.9% Indian, 25.9% Others	Unweighted: 28.4% (57) Secondary, 71.6% (144) Tertiary<br><br>Weighted by ethnicity: 26.6% (54) Secondary, 73.4% (150) Tertiary	Unweighted: 62.7% (126) Bottom 40 (<RM4387), 24.9% (50) Middle 40 (RM4387-<RM9695), 12.4% (25) Top 20 (>RM12586)<br><br>Weighted by ethnicity: 68.5% (140) Bottom 40 (<RM4387), 19.3% (40) Middle 40 (RM4387-<RM9695), 12.2% (25) Top 20 (>RM12586)	NA
Sierra 2021	31	Sex: 100% female	Mean = 52 years	NR	41.9% university or higher degree; 12.9% high school certificate or trade/apprenticeship; 22.6% no school	NR	NR

					certificate or other qualifications; 22.6% unknown.		
Turner 2016	700	Sex: 100% male	Mean = 45 years	100% White	45% college graduates	46% had annual income of $50,000-$100,000	NR
Veron 2018	448	Sex: 100% female	Mean = 51 years	NR	56% postgraduate education	NR	NR
Veron 2023	141	Sex: 100% female	Mean = 40.5, range 32.2-49	NR	81% had tertiary education	NR	NR
Woof 2020	19	Sex: 100% female	26.3% under 50 years old, 63.2% were of screening age (50+), 10.5% did not disclose age	100% Pakistani	NR	Not reported but all lived in highly deprived areas of East Lancashire, UK	NR
Wu 2013	1184	Gender: 58.6% female	Mean (SD) = 58.8 (11.8)	81.8% White, 13.5% Black, 4.7% other	13.3% high school or less, 20.7% some college, 38.9% college degree, 27% any graduate	NR	NR

[a]Of the 42 studies, 36 studies included only general public participants in their sample and 6 studies included both general public participants and healthcare professionals.
[b]We followed the original study's reporting of whether they measured sex or gender and the categories that they reported. If the study did not report whether they measured sex or gender, we only reported the categories that they used.

Table F2: Characteristics of health-care professional (HCPs) participants (N=2,289, 27 studies[a]).

Source	Sample Size	Sex/Gender[b]	Age (Years)	Profession and Specialty	Level of experience
Blouin-Bougie 2021	15	Gender: 93.3% women	NR	6 MD general practitioners, 5 MD specialists, and 4 genetic counsellors	NR
Braithwaite 2023	21	Gender: 57.1% female	Mean (SD) = 51.5 (10)	17 (81.0%) Family medicine, 3 (14.3%) General internal medicine, 1 (4.7%) Other	NA
Conley 2024	72 (72 completed surveys, of which 17 participated in interviews)	Gender: Surveys = 69% women; Interviews = 59% women	NA	Surveys: 56% Primary Care, 22% Gynecology, 22% Radiology, 19% General radiology, 81% Breast radiology; Interviews: 47% Primary Care, 24% Gynecology, 29% Radiology, 20% General radiology, 80% Breast radiology	Surveys: 32% <10 years, 32% 11-20 years, 36% 21+ years. Interviews: 41% <10 years, 24% 11-20 years, 35% 21+ years
Dodd 2024	84	Gender: 58.5% female	43.9% aged 18-40, 45.1% aged 41-60, 11% aged 61 and above	15.9% were general practitioners, 13.4% nurses, 12.2% radiation oncologists, 11% radiologists, 7.3% policy/program managers, 4.9% medical oncologists, 3.7% allied health professional, 2.4% researchers, 1.2% trainee or general practitioner registrar, 17.1% other	37.8% had 0-10 years of professional experience, 20.7% had 11-20 years, 25.6% had 21-30 years, 14.6% had 30+ years, and 1.2% not applicable
DuBenske 2021	11	90.9% female	NR	All primary care physicians	9-36 years in practice
French 2022	29	NR	NR	9 Radiographer, 1 mammographer, 5 Consultant Radiologist, 2 Clinical Fellow (Radiology), 2 Breast Screening Office Manager, 2 Advanced Nurse Practitioner, 1 Family History Risk & Prevention Clinic Nurse, 6 Clinical Nurse Specialist (Breast), 2 General Practitioner	NR
Furst 2018	15	NR	NR	NR	NR
Greenberg 2019	16 (14 completed the baseline survey, of which 6 participated in interviews. 2 new participants completed post-surveys)	Gender: 100% female	Mean = 44.9, range 30-65	69% nurse practitioners, 9% physician assistant, 23% certified nurse mid wives	Mean years of planned parenthood experience = 7.5 years, range 1-20
Hawkins 2022	14	NR	NR	21.4% screening officer managers, 14.3% screening program manager, 14.3% nurse (family history clinic), 14.3% doctor (family history clinic), 14.3% doctor/consultant (radiologist), 21.4% mammography/radiographer	NR
Lapointe 2022	593	Gender: 93.5% women	NR	22.3% physicians, 69.7% nurses, 8.0% other. 36.1% were family medicine/primary care, 12.8% were oncology, 51.1% were other (internal medicine, surgery, emergency, palliative care, public health medicine, radiology, and obstetrics-gynecology)	12.5% had <5 years of practice, 29.2% had 5-14 years experience, 24.4% had 15-25 years, 33.9% had >25 years experience.
Lapointe 2023	593	Gender: 93.5% women	NA	36.1% Family medicine/Primary care (n=167), 12.8% Oncology (n=59), Other (includes genetic counsellor; physiotherapist; occupational therapist; medical imaging; researcher and technologist) 51.1% (n=236), Missing data (n=131).	12.5% <5 years, 29.2% 5-14 years, 24.4% 15-25 years, 33.9% >25 years

Laza-Vasquez 2022a	29	NR	NR	NR	NR
Laza-Vasquez 2022b	220	Gender: 76.3% female	Median = 53 (95% CI: 44.8, 60.0)	19.5% oncology; 19% Epidemiology/Preventive Medicine and Public Health; 14.9% Family and Community Medicine; 10.8% No specialty, 12.8% Gynecology and Obstetrics; 3.6% Radiology; 3.1% Health economics; 1.5% Surgery; 14.9% Other.	Median years of work experience = 25 (95% CI: 16.0, 33.0)
Lowery 2022	105	NR	NR	NR	NR
Matthias 2020	15	Gender: 47% women	Mean (SD) = 46.5 (9.3)	All primary care providers	NR
McWilliams 2020	17	65% female	NR	6 breast cancer healthcare professionals within radiology, oncology, radiography, nursing and surgery; 6 senior academics: ethics, epidemiology, statistics and health economics; and 5 breast screening programme operations/management professions including user involvement.	NR
Pruitt 2024	34	Gender: 62% female	NA	38% Internal medicine, 47% Family medicine, 15% gynecology	23% <5 years, 12% 6-10 years, 15% 11-15 years, 18% 16-20 years, 32% >20 years
Puzhko 2019	11	NR	NR	NR (participants were family physicians or genetic counselors)	NR
Rainey 2018	44	Gender: Netherlands = 84.2% female; United Kingdom = 75.0% female; Sweden = 66.7% female	Median age by country: Netherlands = 54; UK = 48; Sweden = 45	34.1% researcher; 45.5% clinician; 25% other	Median years of experience: Netherlands = 21; UK = 13; Sweden = 11
Resong 2024	151	Gender: 71% female	NR	53% Physician, 26% Nurse Practitioner, 14% Physician Assistant, 7% Other	NA
Rupert 2013	9	NR	NR	NR	NR
Schroy 2015	66 (9 from interviews, 57 from surveys)	Gender: 56% female	NR	65% general internal medicine, 35% family medicine	37% under 10 years in practice, 40% 10-19 years, 23% 20+ years
Schroy 2016	43	NR	NR	43 primary care providers, including 25 board-certified general internists, 9 board-certified family physicians, and 9 nurse practitioners.	NR
Walker 2017	29	Gender: 75.9% female	Median age: General practitioners (GPs) = 50; Practice nurses = 55; Practice managers = 50	14 GPs, 9 practice nurses, 6 practice mangers	GPs: average 22 years in general practice, 30 hours worked in an average week; Practice nurses: 35 years in general practice; Practice mangers: 2 years in general practice
Weigert 2018	11	NR	NR	All mammographers (radiologic technologists)	NR
Woof 2021	28	NR	NR	3 general practitioner, 9 radiographer/mammographer, 5 advanced practitioner, 3 consultant radiology, 1 trainee mammographer, 1 breast care nurse, 6 admin positions	NR
Wu 2013	14	Gender: 50% female	Range 29-35	9 physicians and 1 nurse practitioner at one site, 4 physicians at another	NR

[a]Of the 27 studies, 21 studies included only HCPs in their sample and 6 studies included both general public participants and HCPs.

[b]We followed the original study's reporting of whether they measured sex or gender and the categories that they reported (e.g., female vs woman). If the study did not report whether they measured sex or gender, we only reported the categories that they used.

Appendix G: Quality appraisal scores of included studies (N=63).

Source	Study Design[a]	Overall Screening Questions (All Study Designs)[b]		Study Design-Specific Questions					Total Score[c]
		Q1	Q2	Q3	Q4	Q5	Q6	Q7	
Bas 2023	1	Yes	Yes	Yes	Yes	Yes	Yes	Yes	7/7
Blouin-Bougie 2021	1	Yes	Yes	Yes	Yes	Yes	Yes	Yes	7/7
Dodd 2024	1	Yes	Yes	Yes	Yes	Yes	Yes	Yes	7/7
French 2022	1	Yes	Yes	Yes	Yes	Yes	Yes	Yes	7/7
Furst 2018	1	No	Yes	Yes	Yes	Yes	Yes	Yes	6/7
Hawkins 2022	1	Yes	Yes	Yes	Yes	Yes	Yes	Yes	7/7
He 2018	1	Yes	Yes	Yes	Yes	Yes	Yes	Yes	7/7
Hindmarch 2023	1	Yes	Yes	Yes	Yes	Yes	No	Yes	6/7
Kelley-Jones 2021	1	Yes	Yes	Yes	Yes	Yes	Yes	Yes	7/7
Laza-Vasquez 2022a	1	Yes	Yes	Yes	Yes	Yes	Yes	Yes	7/7
Lippey 2019	1	Yes	Yes	Yes	Yes	Yes	Yes	Yes	7/7
Matthias 2020	1	Yes	Yes	Yes	Yes	Yes	Yes	Yes	7/7
McWilliams 2020	1	Yes	Yes	Yes	Yes	Yes	Yes	Yes	7/7
McWilliams 2021	1	Yes	Yes	Yes	Yes	Yes	Yes	Yes	7/7
McWilliams 2023	1	Yes	Yes	Yes	Yes	Yes	Yes	Yes	7/7
Rainey 2019	1	Yes	Yes	Yes	Yes	Yes	Yes	Yes	7/7
Rainey 2020a	1	Yes	Yes	Yes	Yes	Yes	Yes	Yes	7/7
Roberts 2021	1	Yes	Yes	Yes	Yes	Yes	Yes	Yes	7/7
Sierra 2021	1	Yes	Yes	Yes	Yes	Yes	Yes	Yes	7/7
Walker 2017	1	Yes	Yes	Yes	Yes	Yes	Yes	Yes	7/7
Woof 2020	1	Yes	Yes	Yes	Yes	Yes	Yes	Yes	7/7
Woof 2021	1	Yes	Yes	Yes	Yes	Yes	Yes	Yes	7/7
Rainey 2020b	2	Yes	Yes	Yes	Yes	Yes	Yes	Yes	7/7
Schroy 2016	2	Yes	Yes	Yes	Yes	Yes	Yes	Yes	7/7

Schroy 2011	2	Yes	Yes	Yes	Yes	Yes	Yes	Yes	7/7
Turner 2016	2	Yes	Yes	Yes	Yes	Yes	Yes	Yes	7/7
Braithwaite 2023	3	Yes	Yes	No	Yes	Yes	Yes	Yes	6/7
French 2018	3	Yes	Yes	Yes	Yes	Yes	Yes	Yes	7/7
Ghanouni 2020b	3	Yes	Yes	Yes	Yes	Yes	Yes	Yes	7/7
Lapointe 2022	3	Yes	Yes	Yes	Yes	Yes	Yes	Yes	7/7
Lippey 2023	3	Yes	Yes	No	Yes	Yes	Can't tell	Yes	5/7
Miller 2021	3	Yes	Yes	Yes	Yes	Yes	Can't tell	Can't tell	5/7
Rupert 2013	3	Yes	Yes	Yes	Yes	Yes	Can't tell	Yes	6/7
Veron 2023[d]	3	Yes	Yes	Can’t tell	Yes	No	Can’t tell	Yes	4/7
Ghanouni 2020a	4	Yes	Yes	Yes	Yes	Yes	Can't tell	Yes	6/7
Koitsalu 2016	4	Yes	Yes	Yes	Yes	Yes	Yes	Yes	7/7
Koitsalu 2021	4	Yes	Yes	Yes	Can't tell	Yes	Can't tell	Yes	5/7
Lapointe 2023	4	Yes	Yes	Yes	No	Yes	Yes	Yes	6/7
Lau 2015	4	Yes	Yes	Yes	Can't tell	Yes	Can't tell	Yes	5/7
Laza-Vasquez 2022b	4	Yes	Yes	Yes	No	Yes	No	Yes	5/7
Loft 2024	4	Yes	Yes	Yes	Can’t tell	Yes	Yes	Yes	6/7
Lipkus 2018	4	Yes	Yes	Yes	Yes	Yes	Can't tell	Yes	6/7
Mbuya-Bienge 2021	4	Yes	Yes	Yes	Yes	Yes	Yes	Yes	7/7
Meisel 2015	4	Yes	Yes	Yes	Yes	Yes	Can't tell	Yes	6/7
Morman 2017	4	Yes	Yes	Yes	Yes	Yes	Yes	Yes	7/7
Piper 2018	4	Yes	Yes	Yes	Can't tell	Yes	Can't tell	Yes	5/7
Pruitt 2024	4	Yes	Yes	Yes	Can't tell	Yes	Can't tell	Yes	5/7
Rainey 2022	4	Yes	Yes	Yes	Yes	Yes	Yes	Yes	7/7
Resong 2024	4	Yes	Yes	Yes	Can't tell	Yes	No	Yes	5/7
Segar 2024	4	Yes	Yes	Yes	Yes	Yes	No	Yes	6/7
Veron 2018[d]	4	Yes	Can't tell	Can't tell	Can't tell	Can't tell	Can't tell	Can't tell	1/7
Weigert 2018	4	Yes	Yes	Can't tell	Can't tell	Yes	Can't tell	Yes	4/7
Wu 2013	4	Yes	Yes	Yes	Can't tell	Yes	Can't tell	Yes	5/7

Bancroft 2015	5	Yes	Yes	Yes	Yes	Yes	Yes	Yes	7/7
Conley 2024	5	Yes	Yes	Yes	Yes	Yes	Yes	Yes	7/7
DuBenske 2021	5	Yes	Yes	Yes	Yes	Yes	Yes	Yes	7/7
Greenberg 2019	5	Yes	Yes	Yes	Yes	Yes	Can't tell	Yes	6/7
Han 2019	5	Yes	Yes	Yes	Yes	Yes	Can't tell	Can't tell	5/7
Liow 2022	5	Yes	Yes	Yes	Yes	Yes	Can't tell	Yes	6/7
Lowery 2022	5	Yes	Yes	Yes	Yes	Yes	Yes	Yes	7/7
Puzhko 2019	5	Yes	Yes	Can't tell	Yes	Yes	Yes	Yes	6/7
Rainey 2018	5	Yes	Yes	Yes	Yes	Yes	Yes	Can't tell	6/7
Schroy 2015	5	Yes	Yes	Yes	Yes	Yes	Yes	Yes	7/7

[a]Study Design: 1=Qualitative (n=22); 2=Quantitative RCT (n=4); 3=Quantitative Non-Randomized (n=8); 4=Quantitative Descriptive (n=19); 5=Mixed Methods (n=10).
[b]See details of the Mixed Methods Appraisal Tool (MMAT) in Appendix B.
[c]The responses are recoded (Yes=1; No/Can't Tell=0) and summed up to form a total quality score for each included article.
[d]Only the abstracts were found for these studies. No follow-up full-text articles were found

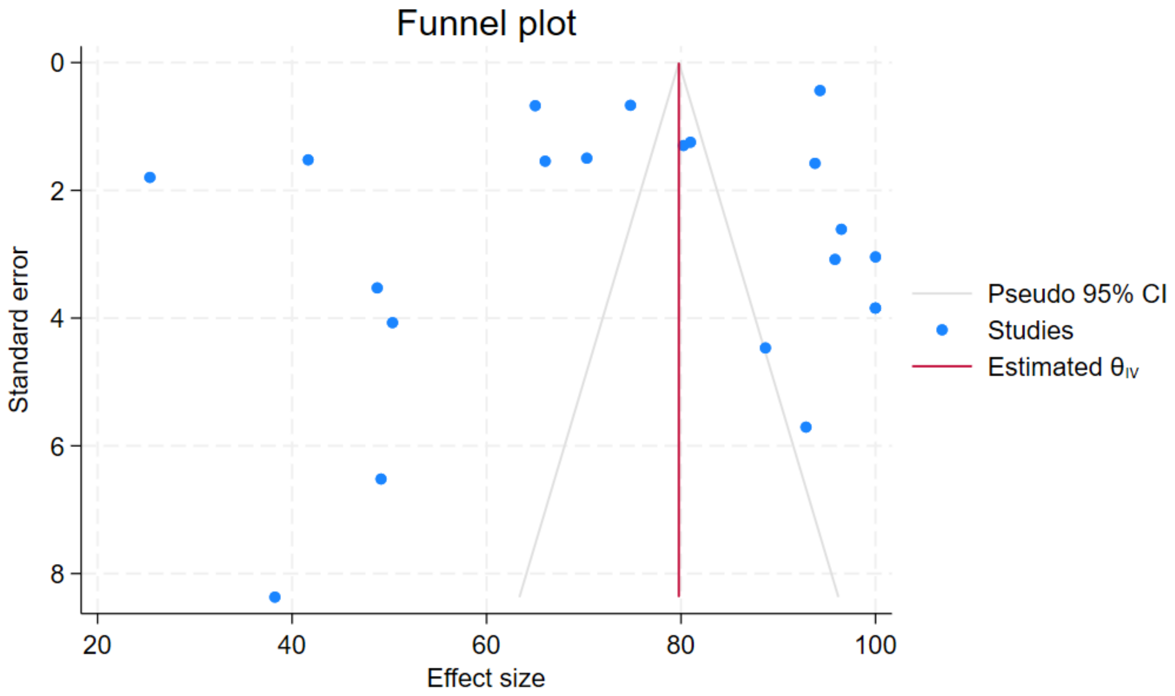

Figure G1: Funnel plot to examine publication bias among included studies.

Appendix H: Summary of themes extracted from qualitative and mixed method studies.

Source	Cancer Site	Population	Themes
Bancroft 2015	Prostate	General public participants	Themes were 1) feeling at risk, 2) living with risk, 3) screening anxiety: the 'great void of uncertainty'
Blouin-Bougie 2021	Breast	Health care professionals (HCPs)	Findings were organized by 11 themes: 1) eligible participants, 2) identification and invitation, 3) risk assessment, 4) risk communication, 5) risk management, 6) ethical approach, 7) services organization, 8) knowledge management, 9) human resources administration, 10) patients or population, and 11) services delivery.
DuBenske 2021	Breast	HCPs; General public participants	No qualitative themes, findings organized under quantitative findings.
French 2022	Breast	HCPs	Three themes were identified: 1) service constraints (limited capacity within current breast services and concerns about the impact of additional workload), 2) risk communication (concerns about optimal way to convey risk to women within resource constraints), and 3) accentuating inequity (how risk stratification could decrease screening uptake for underserved groups).
Furst 2018	Breast	HCPs	Themes included assessments of 1) individualized screening, 2) of women's need for counseling in mammography screening 2.0, 3) of doctors' counseling competence, and 4) of implementation of individualized screening.
Greenberg 2019	Breast	HCPs	Results from the 2 surveys and the interviews were synthesized: 1) participants report Breast Risk Stratification Questionnaire (BRSQ) utilization with intended frequency, 2) clinicians more confident in administering and discussing BRSQ results, less confident discussing genetic services, 3) clinicians believe genetic services are valuable but are uncertain about financial accessibility, 4) clinicians report knowledge gaps regarding cancer genetics and genetic counseling, and 4) execution of educational modules resulted in increased knowledge.
Han 2019	Lung	General public participants	Three qualitative themes were identified: 1) disbelief of personalized cancer risk information, 2) uncertainty about personalized cancer risk information, 3) lack of influence of personalized risk information.
He 2018	Breast	General public participants	Three themes were identified 1) views of traditional screening, 2) views of risk-based mammography screening, 3) women's acceptability of risk-based mammography screening.
Kelley-Jones 2021	Breast	General public participants	Five themes were identified: 1) risk perceptions and acceptability of personalized risk assessment, 2) ways of responding to risk-stratified breast screening scenarios, 3) influence of 'ladder of risk' on responses to scenarios, 4) concerns and conditions of acceptability, and 5) perceived effectiveness.
Laza-Vasquez 2022a	Breast	HCPs	Themes identified were barriers and facilitators related to 1) women participating in personalized risk-based breast cancer screening, 2) health professionals being involved in personalized risk-based breast cancer screening, 3) implementing a risk-based screening program, and 4) health system implementation. The final theme was organizational proposals for implementation of a personalized breast cancer screening program.
Lippey 2019	Breast	General public participants	There were six themes identified: 1) Attitudes and values toward the current breast screening model; 2) Experiences of false positives detected by breast screening; 3) Attitudes toward individualized breast screening; 4) Reservations about individualized screening; 5) Attitudes toward genetic testing; and 6) Perceptions about changing the screening frequency.
Lowery 2022	Lung	HCPs	No qualitative themes, findings organized under quantitative findings or quotations incorporated in discussion.
Matthias 2020	Colorectal	HCPs; General public participants	Four facilitators were identified: 1) use of risk tools is consistent with current practice, 2) the tool has potential to increase screening rates, 3) the tool could lead to improved patient safety and resource allocation, and 4) tool could facilitate discussion about CRC screening. Three barriers were identified: 1) skepticism about tool's accuracy, 2) consistency with guidelines, 3) not having time to use the tool.
McWilliams 2020	Breast	HCPs	Three themes were identified: 1) producing the evidence defining low-risk, 2) impact of risk stratification on women is complicated, and 3) practically implementing a low-risk pathway.
McWilliams 2021	Breast	General public participants	Three themes were identified: 1) a good opportunity to receive risk estimation, 2) multi-faceted acceptability of extended screening intervals, and 3) passive approval versus informed choice.

Puzhko 2019	Breast	HCPs	Findings were organized by two broad themes: 1) Implementation of the program (of which there were 7 subthemes, e.g. Introduction of the program and access to screening, Uncertainty about the difference between risk assessment and screening for disease), and 2) Benefits of the program (subthemes were benefits for health professionals and benefits for women).
Rainey 2018	Breast	HCPs	Clustering maps were created for participants from each country (Netherlands, UK, Sweden) to identify themes that best fit the data. Across the 3 countries, the five themes prioritized by professionals were: 1) Anxiety/worry, 2) proactive approach, 3) reassurance, 4) lack of knowledge, 5) organization of risk assessment and feedback.
Rainey 2019	Breast	General public participants	The themes were: 1) Impact of knowledge (Netherlands [NL], Sweden [SE], and United Kingdom [UK]); 2) Belief in science (NL, SE, and UK); 3) Emotional impact (NL and UK); 4) Decision making (SE and UK); 5) Attitude to medicine (NL and UK).
Rainey 2020a	Breast	General public participants	Findings were organized by three themes: 1) risk communication, 2) accessibility of risk-based screening and prevention, and 3) information needs.
Roberts 2022	Lung	General public participants	Four themes were identified: 1) risk of lung cancer death, 2) risk of a false positive, 3) needs and recommendations, and 4) preferences for decision making.
Schroy 2015	Colorectal	HCPs	No qualitative themes, findings organized under quantitative findings.
Sierra 2021	Breast	General public participants	Three themes were identified: 1) Overall attitude toward the polygenic breast cancer risk assessments (PBCRA), 2) Perceived barriers to PBCRA, and 3) Views on the practicalities of PBCRA implementation.
Walker 2017	Colorectal	HCPs	Interview findings were describing using Normalization Process Theory (NPT): 1) coherence of the tool, 2) cognitive participation, 3) collective action, and 4) reflexive monitoring.
Woof 2020	Breast	General public participants	Three themes were identified: 1) attitudes towards risk awareness, 2) anticipated barriers to acceptability, and 3) acceptability of risk communication strategy.
Woof 2021	Breast	HCPs	Three themes were identified: 1) reservations concerning introduction of less frequent screening, 2) considerations for the management of public knowledge, and 3) deliberating service implications and reconfiguration management.
Wu 2013	Breast; Colorectal	HCPs; General public participants	The themes were: 1) Ease of use of MeTree; 2) Satisfaction with MeTree; 2) Impressions of MeTree at 3 months

Appendix I: Findings extracted from quantitative studies and mixed method studies.

Table I1: Measurements identified from participant-focused quantitative (n=21) and mixed methods studies (n=2)[a].

					**Behavioural modifications in response to personalised risk assessment (PRA), categorized by risk level**		
**Source**	**Acceptability: Measure of participant approval of screening programs.**	**Willingness: Measure of willingness to engage with risk assessment.**	**Satisfaction: Participants' contentment with the screening process and overall experience.**	**Intended adherence: Extent of following recommended screening protocols**	**Behaviour outcome**	**High risk**	**Low risk**
DuBenske 2021	NA	NA	88% (n=53)[b]	NA	NA	NA	NA
French 2018	NA	NA	22.99[c] (n=191) T-C only<br><br>22.91[c] (n=252) T-C + SNP	4.67[d] (n=189) T-C only<br><br>4.76[d] (n=252) T-C + SNP	NA	NA	NA
Ghanouni 2020a	64%[e] (n=933)	NA	NA	NA	Willingness to adapt screening frequency based on risk	89% (n=933)	51% (n=933)
Ghanouni 2020b	25%[f] (n=587)	NA	68% [g] (n=149) benefit<br><br>18% [g] (n=149) risk	NA	NA	NA	NA
Koitsalu 2016	96%[h] (n=1438) of men<br><br>97%[i] (n=1384) of women	95%[j] (n=1438) of men<br><br>94%[j] (n=1384) of women	NA	NA	Willingness to adapt screening frequency based on risk	97% (n=1438) of men<br><br>98% (n=1384) of women	83% (n=1438) of men<br><br>64% (n=1384) of women
Koitsalu 2021	NA	NA	NA	NA	Intention to screen[k]	1.3 (n=391) baseline<br><br>1.2 (n=377) follow-up	1.3 (n=83) baseline<br><br>1.1 (n=85) follow-up
Lau 2015	97%[l] (n=58)	NA	(n=60)<br>46.33[m] Before<br><br>15.08[m] After	NA	Preference to screen	Before<br>70% (n=10)<br><br>After<br>90% (n=10)	Before<br>63% (n=49)<br><br>After<br>40% (n=49)
Lipkus 2018	4.6[n] (n=560)<br><br>4.0[o] (n=560)	NA	NA	NA	Intention to screen	r = 0.45[p] ($p <$ 0.0001)	NA
Lippey 2023	94%[q] (n=241)	NA	NA	NA	Acceptability of varying	Pre-DA (n=127) 99%[r]	Pre-DA (n=127) 72%[s] low risk

					screening intervals		43%[t] much lower risk
Liow 2022	81%[u] (n=993)	NA	NA	NA	NA	NA	NA
Loft 2024	74%[v] (n=5001)	65%[w] (n=5001)	NA	NA	Willingness to adapt screening frequency	89%[x] (n=5001)	42%[y] (n=5001)
Mbuya-Bienge 2021	75%[z] (n=4219)	NA	NA	NA	Willingness to change screening frequency	86% (n=4219)	49% (n=4219)
Meisel 2015	66%[aa] (n=942)	NA	NA	NA	Willingness to adapt screening frequency based on risk	85%[bb] (n=942)	59%[bb] (n=942)
Morman 2017	68%[cc] (n=69)	NA	NA	NA	NA	NA	NA
Piper 2018	42%[dd] (n=1049)	NA	NA	NA	NA	NA	NA
Rainey 2020b	NA	80%[ee] (n=942)	NA	NA	Acceptance of recommended Screening Interval	31%[ff] (n=99) frequency<br><br>65%[gg] (n=101) start age	31%[hh] (n=229) frequency<br><br>39%[ii] (n=229) start age
Rainey 2022	NA	NA	NA	95%[jj] (n=325)	Acceptance of recommended Screening Interval	65%[kk] (n=60)	40%[ll] (n=60)
Rupert 2013	NA	96%[mm] (n=48)	79%[nn] (n=48)	NA	NA	NA	NA
Schroy 2011	NA	NA	50.5[oo] (n=214)	NA	NA	NA	NA
Segar 2024	63%[pp] (n=201) effectiveness<br><br>97%[qq] (n=205) importance<br><br>73%[rr] (n=205) good idea	NA	NA	NA	Willingness to adapt screening frequency	85%[ss] (n=205)	69%[tt] (n=204) average risk<br><br>38%[uu] (n=205) much lower than average risk
Turner 2016	NA	NA	NA	4%[vv] (n=336)<br><br>35%[ww] (n=259)	NA	NA	NA
Veron 2023	NA	NA	98%[xx] (n=141)	NA	NA	NA	NA

[a] Bancroft 2015, Han 2019, Braithwaite 2023, Miller 2021, Veron 2018, and Wu 2013 were not included in this table as they did not have outcomes relevant to this table.

[b] In response to "How valuable was the BCARE experience to you?" most women found BCARE-DA somewhat (32%), very (35%), or extremely (21%) valuable.

[c] Mean satisfaction with the information was assessed using four items ($\alpha = 0.86$), adapted from a previously published scale, which asked women how clear they found the information, how confusing they found it, how well informed they feel about their breast cancer risk information and how satisfied they with the amount of information given. Response options were 'strongly disagree', 'disagree', 'disagree somewhat', 'undecided', 'somewhat agree', 'agree' and 'strongly agree'; Tyrer–Cuzick (T-C) model; T-C including breast density plus single-nucleotide polymorphisms (SNPs)

[d] Mean intention to attend next mammogram assess using a 5-point scale with responses 'strongly disagree', 'disagree', 'neither agree nor disagree', 'agree', 'strongly agree'); Tyrer–Cuzick (T-C) model; T-C including breast density plus single-nucleotide polymorphisms (SNPs)

[e] Participants were asked what they thought 'of the idea of using information like age, family history, reproductive history, lifestyle factors, weight, and results from genetic testing to identify women who are at very high or very low risk of developing breast cancer' ('very bad idea', 'bad idea', 'good idea', 'very good idea', 'not sure', 'prefer not to say').
[f] Preference for risk-stratified screening over status quo
[g] Perceived benefits and risks of screening were assessed using a scale with response options from 0 to 100, denoting how positively (for benefits) or negatively (for risks) participants rated these. For benefit of RBS, respondents selected a value above 74 on a scale from 0 to 100. For risk of RBS, respondents selected between 48 and 100.
[h] If men would participate in risk-based screening
[I] If women would participate in a risk-based breast cancer screening program despite frequency.
[j] Willingness to know their risk
[k] Intention to screen assess on a 5-point scale
[l] Found the decision aid useful in helping them come to an LCS decision
[m] Decisional conflict score
[n] Mean score of a seven-point Likert scale from 1 = Not at all useful to 7 = Extremely useful to assess usefulness of risk assessment
[o] Mean score of a six -point Likert scale from 1 = Extremely inaccurate to 6 = Extremely accurate to assess perceived accuracy of risk assessment
[p] Correlation between higher composite risk appraisal scores and screening intention
[q] Definitely or probability interested in PRBS
[r] Willingness to increase screening frequency from every year
[s] Willingness to reduce screening frequency to every 3 years if low risk
[t] Willingness to reduce screening frequency to every 5 years if much lower risk
[u] Strongly agreed or agreed to expressing positive emotions about receiving their risk assessment reports
[v] Thought it was a good idea to use personal risk to determine mammography screening frequency
[w] Wanted to know personal risk
[x] Would increase screening frequency from every 2 years to every year
[y] Would reduce screening frequency from every 2 years to 4 years
[z] Comfortable providing information for risk assessment
[aa] Good/very good idea (vs. bad/very bad idea/not sure) to vary frequency of BC screening by risk
[bb] Happy/very happy (vs. very unhappy/unhappy/not sure) to have more/less frequent BC screening if at higher/lower risk
[cc] BRCA (high-risk women only) was very helpful or somewhat helpful
[dd] Responses of 5-7 on 7-point scale that assessed if it was reasonable to use a risk calculator to determine when to stop screening
[ee] Desire to know risk
[ff] Screen more frequently than once per year
[gg] Start screening at age 40-45
[hh] Screen less frequently than every 2 years
[ii] Start screening after age 50
[jj] Adhered to risk-based screening recommendation
[kk] Screen more than recommended 18-month interval
[ll] Screen less than recommended 3-year interval
[mm] Chose to calculate risk
[nn] Somewhat or very satisfied with the tool
[oo] Overall satisfaction with Decision Aid + Risk assessment (Mean Decision-Making Process Score; maximum score = 60)
[pp] PRBS was more effective than age-based screening
[qq] PRBS was important
[rr] PRBS was a good idea
[ss] Would be willing to increase screening frequency from every 3 years to 2 years
[tt] Would be willing screen less than every 2-3 years if average risk.
[uu] Would be willing to not be offered any screening if much lower-than-average risk
[vv] Engaged in PSA screening per self-report during 3-month follow-up.
[ww] Engaged in PSA screening, per medical record review, during 3 years of follow-up in the genetic risk score arm
[xx] Satisfied with the screening program

Table I2: Measurements identified from health-care professional (HCP)-focused quantitative (n=8) and mixed methods studies (n=4[a]).

	Individual-level			System-level	
**Source**	**Acceptability**	**Utilisation**	**Self-efficacy: Belief in their ability to execute PRBS**	**Barriers to implementation**	**Facilitators to implementation**
DuBenske 2021	100% (n=11) BCARE-DA facilitated SDM about mammography with their patients	NA	NA	r = 0.347, p = 0.011 (n=11) correlation between length of discussion with patient and estimation of effort required[b]	NA
Greenberg 2019	100% (n=14) administered BRSQ at least once per week	NA	4.5 (SD = 0.52, n=14) mean confidence in administering BRSQ out of 5<br><br>4.43 (SD = 0.67, n=14) mean confidence in interpretation out of 5	NA	NA
Lapointe 2023	n=593[c]<br>High-risk patients:<br>88% increase screening frequency, 88% start screening earlier<br><br>Lower than average risk patients:<br>35% decrease screening frequency<br><br>Much lower than average risk patients:<br>9% do not offer screening, 31% delay start of screening	NA	n=593[d]<br>Discuss advanced and limitations (55%), collect patient information (74%), discuss results (57%), explain risk of developing cancer in the future and having a diagnosis (66%)	NA	n=593[e]<br>Access and inclusion:<br>Access to PCP (16%), access to nurse or NP (11%), access to screening (12%)<br><br>Resources:<br>Number of NPs (13%), number of PCPs (8%), number of genetic counsellors (8%), renumeration of HCPs (2%)<br><br>Time:<br>Time allocated for patient-physician appointment (11%)<br><br>Training and knowledge:<br>Medical training (10%)
Lapointe 2022	NA	NA	7% (n=593) strongly agree or agree that they have enough knowledge<br><br>77% (n=594) strongly agree or agree that they require more training	35% (n=593) strongly disagree or disagree about having ample time to educate themselves	70% (n=593) strongly agree or agree that more education is important (medical curriculum)<br><br>71% (n=593) strongly agree or agree that more education is important (nursing curriculum)
Laza-Vasquez 2022b	85% (n=220) rated the importance of moving from the current screening program to personalized BC screening is important or very important	NA	NA	n=220[f]<br>Resource constraints: HCP workload (71%), limited human resources (70%), limited financial resources (68%)<br><br>Training and knowledge gaps:<br>lack of training (58%), limited knowledge about risk and harms	n=220[f]<br>Patient facilitators:<br>Confidence in HCPs (87%), growing autonomy of patient decision-making (78%), positive perception of HCPs (76%), acceptance of screening program by general population (78%)<br><br>HCP facilitators:

				(50%), Difficulty communicating risk (48%)<br><br>Lack of coordination:<br>private and public HCPs (70%), HCPs at different levels of care (58%), HCP and patient due to social factors (52%)<br><br>Structural resources:<br>Future HCP roles (63%), lack of legal and ethical guidelines (60%), lack of computer support (60%)<br><br>Access and inclusion:<br>Including women without coverage (49%), lack of evidence (39%)<br><br>Resistance to change (41%)	Acceptance of screening program (87%), experience in current screening programs (75%)
Lowery 2022	NA	3.5 (SD = 3.7)[g] Standard implementation<br><br>190.3 (SD = 174.8)[g] 4 original facilities randomized to enhanced implementation (intention to treat)<br><br>224.3 (SD = 197.1)[g] 3 facilities in the enhanced implementation program (as treated)<br><br>246.0 (SD = 164.9)[g] 3 facilities with a full-time screening coordinator engaged in SDM (key resource scenario)	NA	NA	NA
Pruitt 2024	38% (n=34) used risk assessment tool	NA	43% (n=21) not sufficiently familiar with tool	n=21<br>5% do not have time with patients to use tool, 19% results of tool would not change management	NA
Resong 2024	n=151<br>Interested in adopting PRBS (50%)<br><br>Unsure about adopting PRBS and needed more	NA	NA	n=151<br>Insufficient accuracy (17%)<br><br>Time constraints:<br>Concerns regarding time availability (66%)	n=151<br>Patient facilitators:<br>Increased adherence (40%), increase comfort (37%)<br><br>Practice facilitators:

	information about PRBS (40%)			Patient barriers:<br>Patient adherence to recommendations (24%), decreased patient trust in personalized risk (2%)<br><br>Communication:<br>Difficulty conveying risk (23%)	Maximize benefits of screening (62%), minimize harms of screening (35%)<br><br>Access and inclusion:<br>Include more patients (54%)
Schroy 2015	97% (n=57) would consider using an electronic risk index	NA	NA	NA	n=57[f]<br>Time and efficiency:<br>Output includes recommendations (96%), time needed to complete (91%), number of clicks (75%), key variables auto populated (89%)<br><br>Coordination:<br>Integration into workflow (98%), amenable for use by support staff (89%)<br><br>Access and inclusion:<br>Incorporate patient preferences (85%), results are in low literacy and patient-friendly format (91%)<br><br>Regulatory:<br>Medical-legal documentation (70%)
Schroy 2016	Pre-test[h]<br>4.0 (SD=0.7, n=42) usefulness of tool<br><br>Post-test[h]<br>3.9 (SD=0.9, n=32) usefulness of tool	NA	0% (n=173)[h] selected preparedness as the most important factor influencing provider recommendation	NA	Pre-test (n=42)[h]<br>3.2 (SD=1) reduce time to decide screening modality<br>3.8 (SD=0.7) more receptive to patient preferences<br><br>Post-test (n=32)[h]<br>3.6 (SD=1) reduce time to decide screening modality<br>3.8 (SD=1) more receptive to patient preferences<br>3.2 (SD=1) reduces risk of malpractice
Weigert 2018	100% (n=11)[j] reported that the tool was easy to access, easy to operate, had easy-to-ask question, had little effect on time, and was received well by patients	NA	100% (n=11)[j] reported that it was easy to become skilled at using the tool	NA	NA
Wu 2013	n=14<br>93% recommend to peers, 86% tool improved practice, 79% tool made practice easier, 93% output	NA	NA	NA	NA

	report was useful, 0% affected workflow, 0% disagreed with output report				

[a] Conley 2024, Puzhko 2019, Rainey 2018, Braithwaite 2023, Chu 2023, and Rupert 2013 were not included in this table as they did not have outcomes relevant to this table.
[b] The Observer OPTION-5 (23) assesses physician's effort to involve the patient in SDM (0 = no effort to 4 = exemplary effort) with 5 items: (1) identifying need for a decision exists, (2) describing options, (3) information exchange, (4) preference elicitation, and (5) preference integration.
[c] Selected strongly agree or agree regarding screening recommendations related to different patient risk categories
[d] Selected strongly agree or agree regarding attitudes towards comfort with integrating personalized risk into practice
[e] Participants were allowed to select their top three choices
[f] Total n for the percent that marked very important or important for each barrier or facilitator of implementing a risk-based screening program
[g] 6-month mean number of tool uses
[h] Mean scores between 1 (strongly disagree) and 5 (strongly agree)
[i] Group was those who used the decision AID and risk assessment tool
[j] Rated a 4 on a scale of 1 (not acceptable) to 4 (excellent)

Appendix J: Themes identified from qualitative (n=22) and mixed methods studies (n=10) and supporting quotations.

Themes	Sub-Themes	Supporting Quotations
Acceptability and perceptions of personalised risk assessments among the general public (149 excerpts)	1) Perception of personalized risk assessments<br>2) Emotions related to risk-based screening (RBS)<br>3) RBS and risk perception	"All participants unanimously indicated that they would want to know their personal risk of developing breast cancer in the next ten years. There were no participants who were against learning their risk. Women explained that risk information is important for healthcare professionals to share and introducing this service into the NHSBSP would be a good idea in order to raise awareness: Yes, you should do, should give out this. It is a good idea to let people know whereabouts they stand on the risk band. Because anything to do with your health and wellbeing, it's good to have that information available so that you can make improvements and adjustments in order to improve that risk factor. (Fatima, 60, via interpreter)" (Woof et al, 2020)<br><br>"After discussion of several factors which contribute to breast cancer risk, many women expressed concern about change in some risk factors and highlighted the non-static nature of some of the variables. For example, women identified that their lifestyle factors, such as body mass index, alcohol, or exercise, may change significantly over a 5-year period. Many women expressed a preference for their risk to be reassessed at regular timepoints in the program, rather than just a one-off assessment on entry to the program. "..your family history would change quite possibly as your parents get older and brothers and sisters… a lot of things that would be changing like exposure to hormones where you might end up going on HRT but ten years ago you weren't. . .there's a lot of stuff that would need to be readdressed… on a regular type basis" (Participant 12, Focus group 2)" (Lippey et al, 2019)<br><br>"HCPs had qualms about how women would interpret a low-risk estimate. They emphasized that some women could misattribute being at low-risk as having 'no risk', especially if advised to attend screening less often: So people find it difficult to understand risk. So if you say, for example, to a lady, oh, you don't have to come for five years, but they might think, oh, I won't get breast cancer because I'm such a low risk so I won't get it. (Advanced Practitioner – Mammography: FG2)" (Woof et al, 2021)<br><br>"Participants often noted some relief that their absolute risk of lung cancer death was lower than anticipated. One participant remarked, "It's saying in the next five years I don't have much of a chance of dying by this and I have a better chance [of dying in] in a car accident."" (Roberts et al, 2021)<br><br>"Interviewer: So seeing that 8% when you had in your head that it was going to be 75% or even more, did that affect your decision to proceed with getting scanned or tell me about that thinking. Patient: No, no, you know I wasn't looking for a way out of getting a scan. Interviewer: Uh huh. Patient: I wanted the scan. I mean I trust; I mean figures are one thing but I wanted them to get in there and look at my lungs." (Han et al, 2019)
Acceptability and perceptions of personalised risk-based screening among the general public (146 excerpts)	1) Patient acceptability of RBS<br>2) Patient usefulness of RBS<br>3) Perceptions of change in screening frequency<br>4) Patient barriers to RBS<br>5) Patient facilitators to RBS	"Despite very limited awareness of this paradigm for breast cancer, some women reported risk-based screening practices (e.g., low-risk women not being screened every year). Some (n=8) thought the concept of matching screening frequency to personal risk made sense (e.g., I think that this plan makes sense to me… I think it's crazy to treat the whole population like we're all the same and we all have the same risks. We don't.)" (He et al, 2018)<br><br>"It's sort of like, well this is in your DNA, this is what you're lumped with, this is your lot in life. And if you had more information about being able to make better informed decisions, for me it comes down to no regrets. So yeah, that...the more information, the better". (42 years, BRCA2 carrier)" (Sierra et al, 2021)<br><br>"Most women who thought 'more is better' deemed the prospect of 5-year screening intervals unacceptable as this would evoke worry alongside the added burden of taking personal responsibility for checking their breasts: "After the first year of the screen I will worry ... [and will have] to wait another four years. So that means I have to check my body more frequently myself" (12: Regular, 68 years). Several women with 'more is better' responses thought extended screening intervals would be more acceptable if there was an option for elective screening if women are concerned: "There should be maybe an option that even if you are in a low risk ... you should be given the opportunity to have them more frequently" (2: Pre-eligible,41 years). Some women who thought 'more is better' suggested that those who found this

Themes	Sub-Themes	Supporting Quotations
		low-risk scenario acceptable would be tempting fate, with one woman suggesting that “people are going to die” (1: Pre-eligible, 48 years)." (Kelley-Jones et al, 2021)<br><br>"In each group there was at least 1 participant who expressed concern about the motivation for this potential change with suspicion that the change was being driven to save money at the cost of individual health." (Lippey et al, 2019)
Acceptability and perceptions of personalised risk-based screening among health-care professionals (HCPs) (114 excerpts)	1) HCP acceptability of RBS<br>2) HCP usefulness of RBS	"I think screening with mammography has reached its ceiling [. . .] And there is a need for change [. . .] it will no longer be considered beneficial because [. . .] it is already being demonstrated [. . .]. In addition, we have tools that allow us to better estimate risk. [. . .] And you give value to the test and also to the risk estimate. This is very important for adherence! (DG3P1)" (Laza-Vazquez et al, 2022a)<br><br>"The notion of visualization and having a “hard number” to show patients their risk was echoed by a number of PCPs: “If you can show patients a risk score, it can help to persuade them” (PCP404).“Sometimes visuals are more powerful than words”(PCP208).“You could concentrate on people that really need colonoscopies and tell them the statistics, and have some hard data to kind of lead them [to colonoscopy]” (PCP203)." (Matthias et al, 2020)<br><br>"HCPs stressed that confidence in risk estimate accuracy will be essential if low-risk women are to attend screening less frequently. They raised questions regarding the stability of a 10-year risk estimate with concerns about how quickly it could change and subsequently affect the frequency at which women should be screened. They cited the increased risk of breast cancer with age, changes in mammographic density, the inclusion or exclusion of a polygenic risk score and the development of breast cancer in relatives as factors which could change a risk estimate: How do we get around the fact that everybody’s risk increases as they get older? So if you give somebody their risk at 47 when they first come, when is that reviewed? Because we know that the majority of cancers occur in the older cohort of ladies, so your risk just increases as you get older anyway. So do you then at your six or your 12 year mammogram have another density reading and your risk is . . .? (Breast Screening Office Manager: FG1)" (Woof et al, 2021)<br><br>"Participants stated that disengagement in breast screening is a continual problem. They attributed this to women’s personal health anxieties, stress, and reservations about breast screening. Screening staff at all sites were concerned with the balance of meeting screening targets and suggested risk stratification could be an excuse used by women to further reinforce their decision not to attend screening: "I think people that weren’t going to come already have preconceived ideas and already have their excuses…and they will just use this [BC-Predict] for reinforcement if they wanted (Breast Screening Office Manager 2 – site C)" (French et al, 2022)
Barriers and facilitators to personalised risk-based screening implementation among health-care professionals (141 excerpts)	1) HCP barriers to implementing RBS<br>2) HCP facilitators to implementing RBS<br>3) HCP feasibility of implementing RBS	"First, everyone has to be involved, all the actors, primary, specialized, and radiology also thinking in the same way, and then develop a software that maintains it.. which would pose two things: one, how do you approach the population. That is, how do you call them, if you wait for them to come or you call them, and once they have come there is also something that has not been considered; the family doctor sees it, but someone has to organize the agendas, considering that you have this entire population, where one needs an annual mammogram, another every three years, another every two [. . .] In other words, technical offices will be necessary, but we can be an actor who organizes it so that it is primary care who makes the contact, which would be an ideal solution, perfectly trained nurses could do that, and people who simply organize. And don’t worry, I would already be behind for [. . .](DG3P4)" (Laza-Vazquez et al, 2022a)<br><br>"Participants across all sites stated that sufficient funding would need to be made available to support risk stratification. Both radiology and nurse participants acknowledged that identifying and hiring skilled personnel to fulfil instrumental roles to deliver an effective service is already challenging without the added pressure of risk stratified screening "….how is [Minister for Health] gonna get the money together, then, to fund all this? Because it’s difficult, there’s a difficulty recruiting the clinical team, that’s the radiologist, the doctors, the radiographers, all that, very, very difficult (Radiographer 5 – site B)….you can throw money at something but it doesn’t make a difference. You need people with the appropriate skills to be doing the job (Consultant Radiologist 5– site C)" (French et al, 2022)

Themes	Sub-Themes	Supporting Quotations
		"Notably, genetic counsellors mentioned that many health professionals do not sufficiently understand “risk” and that the development of a comprehensive tool to help health professionals understand the meaning of results should be considered. Indeed, consultations with PCPs showed that the participants were unsure how to interpret the 10-year risk for breast cancer based on the communication tools provided by PERSPECTIVE and they were concerned about how to communicate the risks calculated by BOADICEA to women." (Puzhko et al, 2019)<br><br>"Some participants suggested that women from the populations who disengaged from screening were likely to be most at risk of developing breast cancer and would benefit significantly from risk stratification, thus making accessibility imperative: "The women that don’t do the Predict thing [risk assessment], is it because they don’t understand? So are they actually, could they be the group that are more at risk, because o☐ their lifestyle… (Radiographer 5 – site B)" (French et al, 2022)<br><br>"Among the major obstacles to implementation acknowledged by both types of health providers was the lack of time for PCPs during a typical 20–25-min appointment. Many felt that there is simply not enough time to introduce the program, explain risks and benefits of participation, enter the data in BOADICEA, calculate the risks, and explain the test results. As mentioned above, one of the ways to overcome this obstacle suggested by both groups of health professionals was by engaging nurse or other trained personnel for some or all steps of this process. Moreover, if the program becomes a public health matter, then public health nurses could be involved. The use of EMR was mentioned as a means to help facilitate program implementation as the information (or at least part of the information) could be automatically populated from the woman’s medical chart. Finally, integration of the tool in the EMR was also suggested as helping to ensure the routine use of risk stratification tools by PCPs. The participants highlighted that health professionals involved in this process would need to be trained to use the BOADICEA model and other communication tools developed for PERSPECTIVE." (Puzhko et al, 2019)

Appendix K: Additional meta-analysis results.

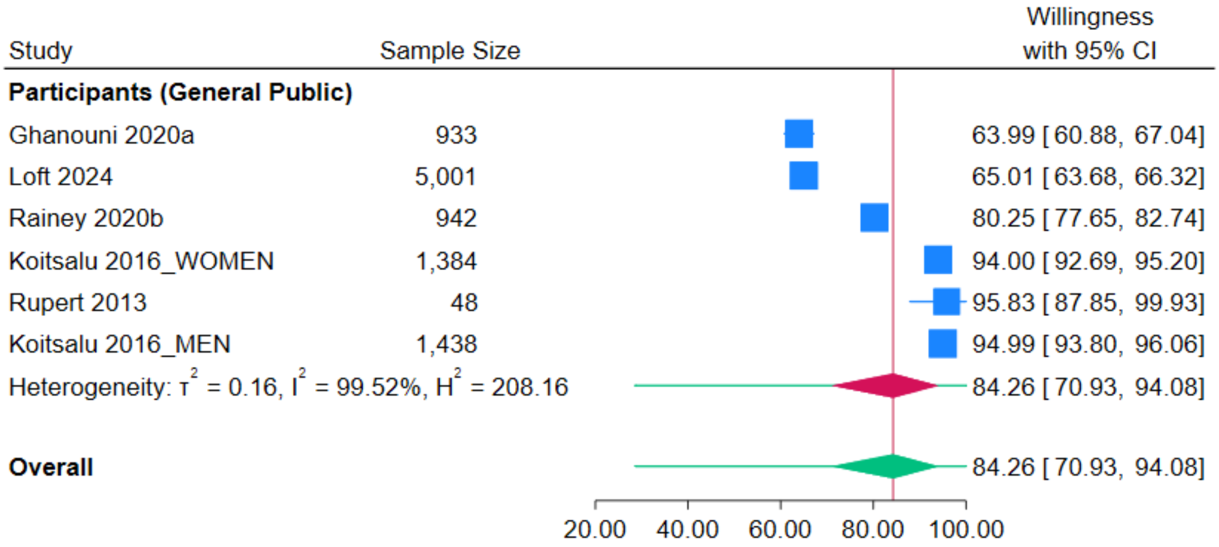

Figure K1: Willingness to engage in personalised risk assessments among general public participants.

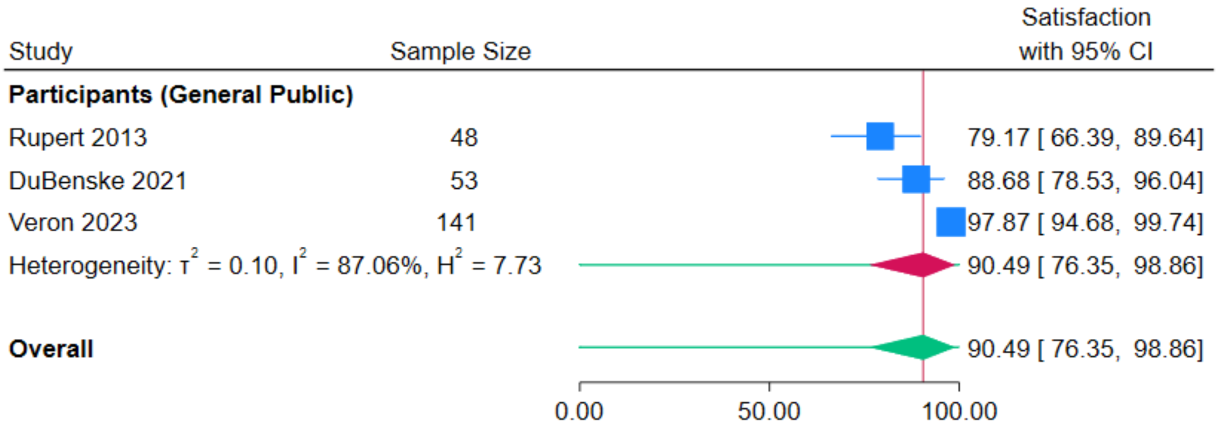

Figure K2: Satisfaction with personalised risk-based screening among general public participants.

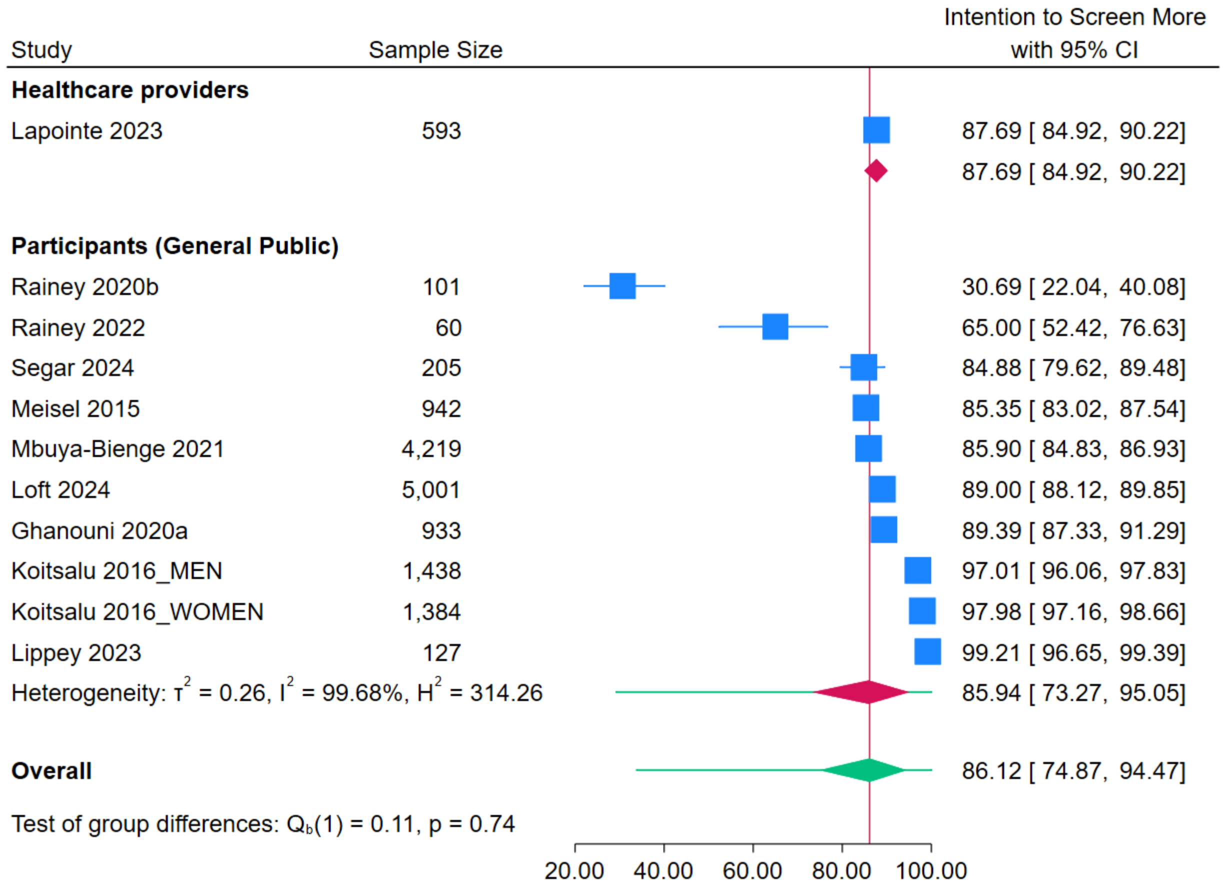

Figure K3: Intentions to screen more based on personalised risk assessments among health-care professionals and general public participants.

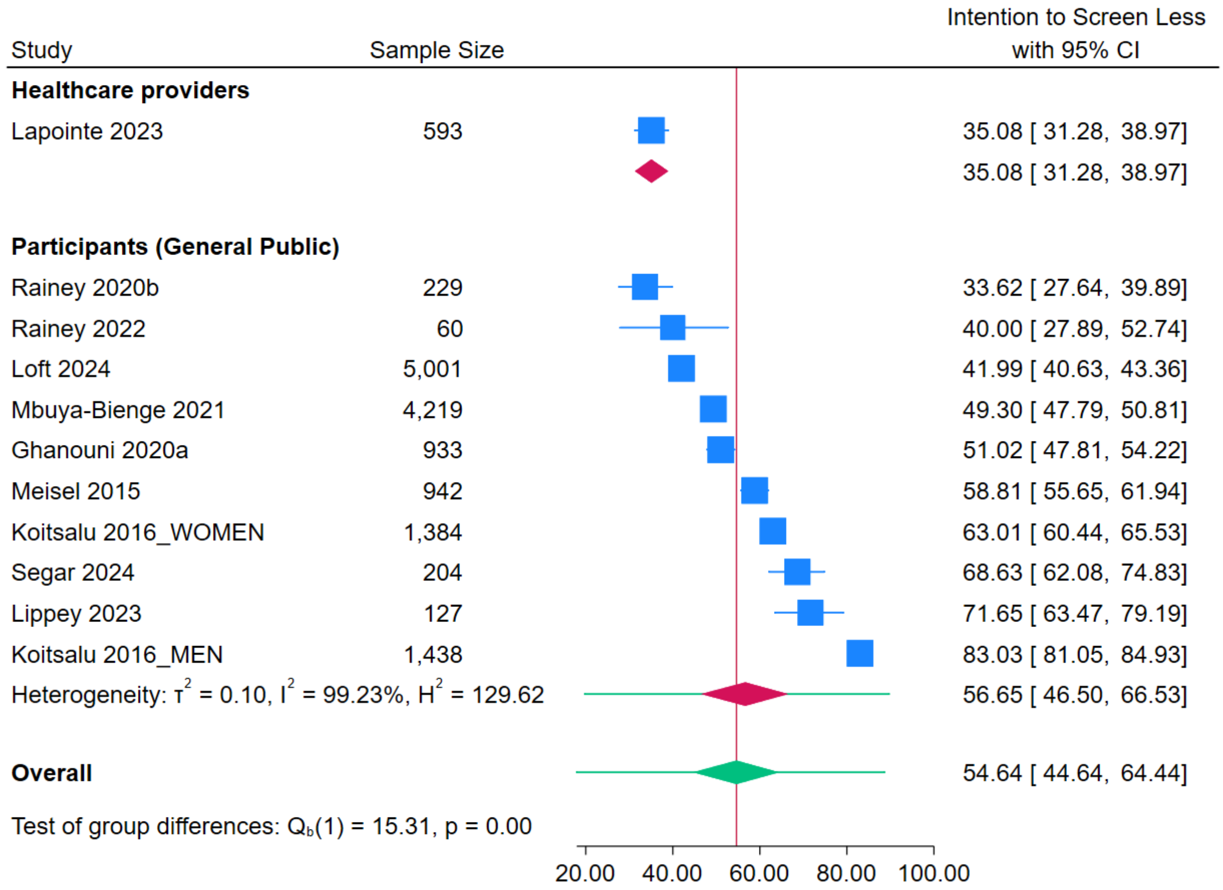

Figure K4: Intentions to screen less based on personalised risk assessments among health-care professionals and general public participants.

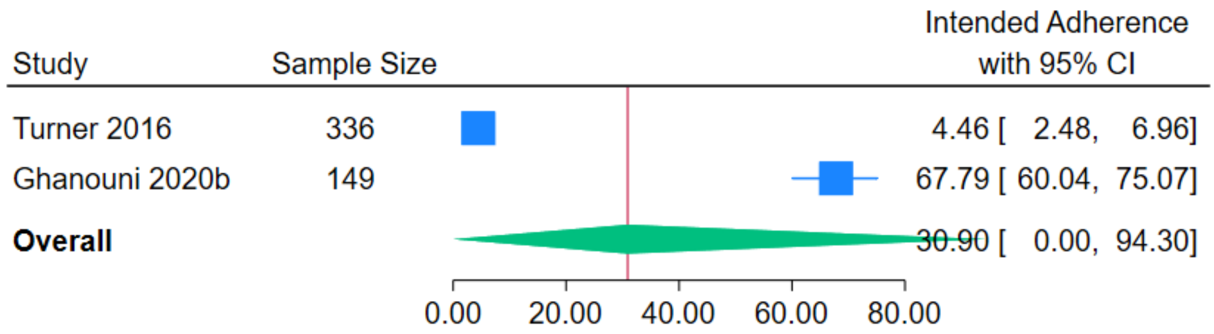

Figure K5: Intended adherence with recommended screening protocol based on personalised risk assessment among general public participants.

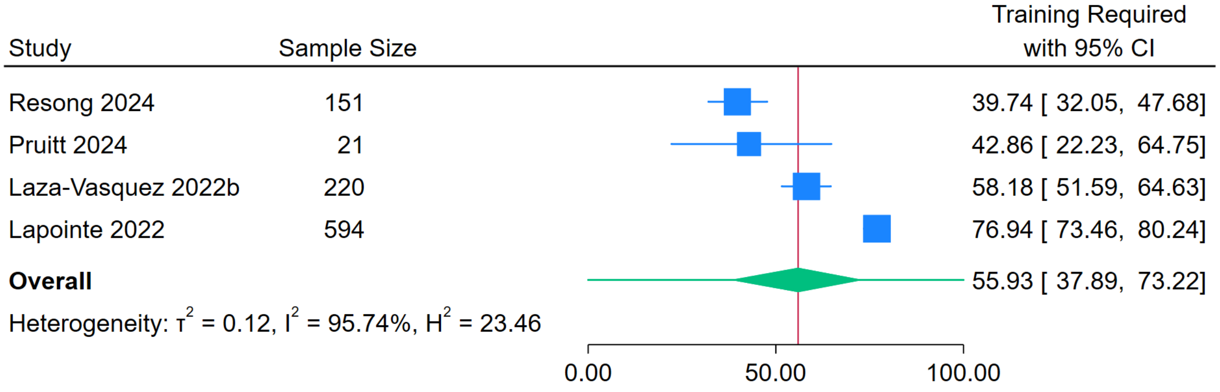

Figure K6: Training required as a barrier to personalised risk-based screening among health-care professionals.

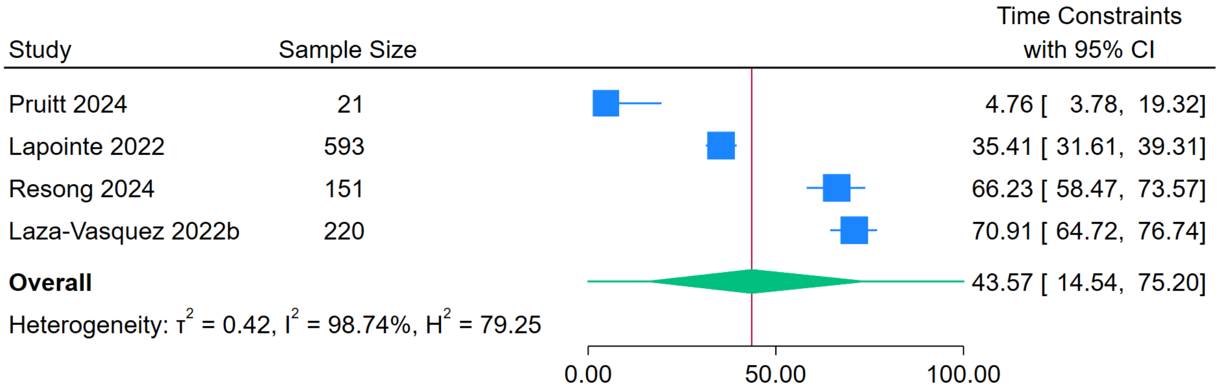

Figure K7: Time constraints as a barrier to personalised risk-based screening among health-care professionals.

Appendix L: Subgroup analyses results.

Subgroup Analysis Results

Among the qualitative findings, we did not find major differences in the findings by cancer site. For cervical cancer screening (one study [Bas 2023]), participants highlighted concerns about sharing their sexual history and HPV infection status for calculating risk, given the sensitivity of this information, which was not raised in other cancer sites. In addition, in the LCS studies, participants more frequently reported that they would not change their risk perceptions for lung cancer or did not believe their PRA due to their smoking history. Compared to other geographic regions, participants from Asia Pacific appeared to have less resistance towards changing their screening pathway based on risk as long as the risk score is explained and a concrete plan for management of risk is provided (Liow 2022). However, this came from only one study in Singapore; hence, we are cautious in generalising this finding. In addition, we did not have any data on PRBS acceptability among HCPs and barriers and facilitators to PRBS implementation among HCPs from Asia Pacific. Finally, in studies from Europe, the impact of the media in shaping public opinion and acceptability of PRBS programs was raised more often compared to studies North America and West Pacific. There were four times as many studies published from 2019-2024 (n=25) compared to studies published from 2010-2019 (n=6), but no major differences in findings were found between these groups, as well as based on participant sex and age. Among the quantitative findings, we observed statistically significant differences in acceptability by cancer site for HCPs, and by cancer site, region, and sex for participants (Figure L1 below). For participants, we also observed subgroup differences for the following outcomes: 1) intended adherence and intention to screen more and less by cancer site and by sex of participants, 2) satisfaction and intention to screen more and less by region; and 3) satisfaction and willingness to engage in PRA by both year of publication and age of participants (Table L1 below).

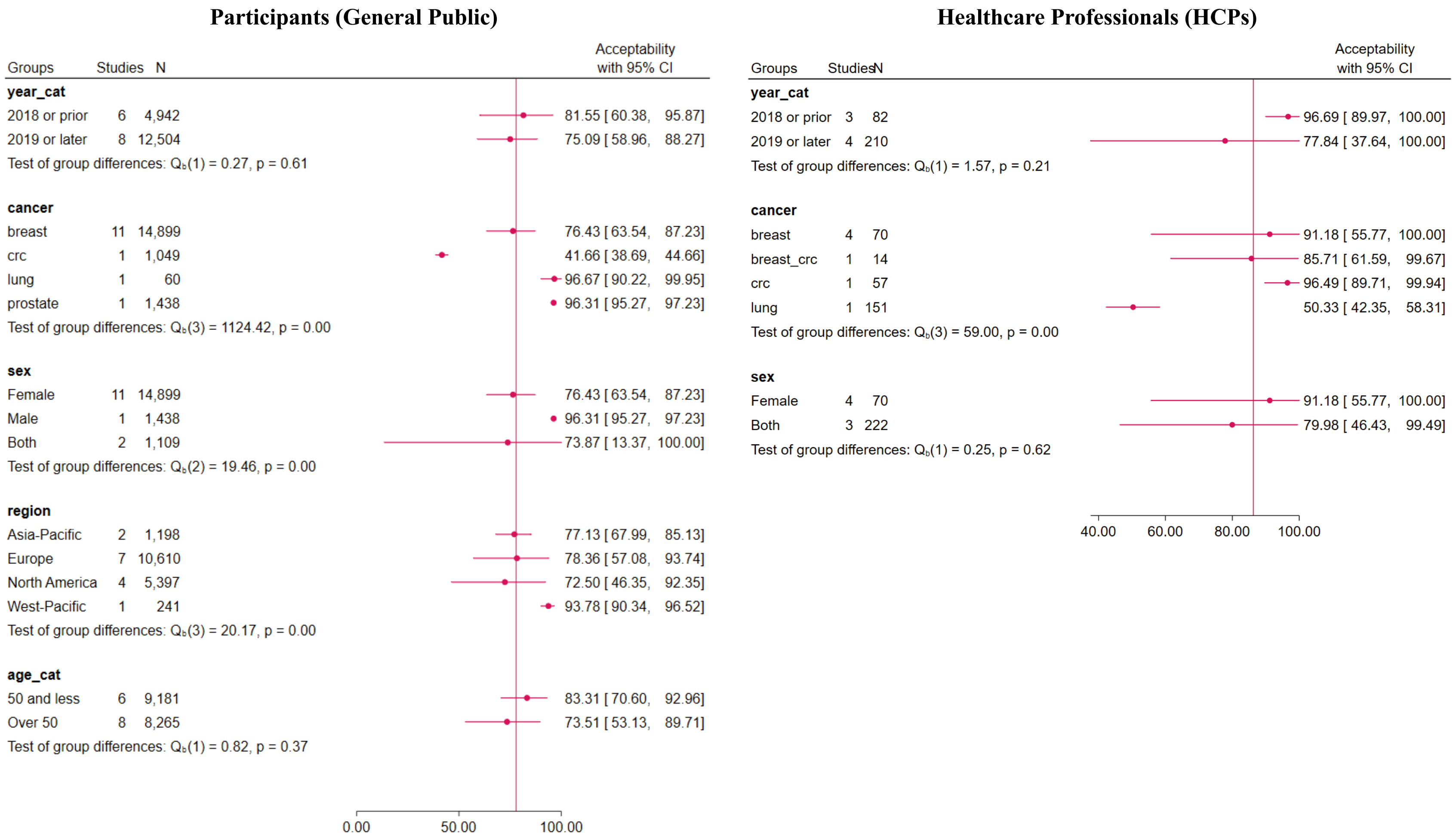

Figure L1. Subgroup analysis of acceptability of personalised risk-based screening among participants (general public) and health-care professionals (HCPs).

Table L1: Meta-analysis results by subgroup.

Outcome	Population	Group	Subgroup	# of studies	Sample size	Percentage (95% CI)	I2
Acceptability of personalised risk-based screening (PRBS)	Participants (General Public)	Year of publication	2019 or later	6	4,942	81.55 (60.38, 95.87)	99.61%
			2018 or prior	8[a]	12,504	70.60 (53.49, 85.16)	99.68%
		Type of cancer*	Breast	11[a]	14,899	76.43 (63.54, 87.23)	99.62%
			CRC	1	1,049	41.66 (38.69, 44.66)	NA
			Lung	1	60	96.67 (90.22, 99.95)	NA
			Prostate	1	1,438	96.31 (95.27, 97.23)	NA
		Sex*	Females	11[a]	14,899	76.43 (63.54, 87.23)	99.62%
			Males	1	1,438	96.31 (95.27, 97.23)	NA
			Both	2	1,109	73.87 (13.37, 100.00)	99.02%
		Region*	Asia-Pacific	2	1,198	77.13 (67.99, 85.13)	86.63%
			Europe	7[a]	10,610	78.36 (57.08, 93.74)	99.80%
			North America	4	5,397	72.50 (46.35, 92.35)	99.46%
			West-Pacific	1	241	93.78 (90.34, 96.52)	NA
		Mean age of participants	50 and less	6[a]	9,181	83.31 (70.60, 92.96)	99.47%
			Over 50	8	8,265	73.51 (53.13, 89.71)	99.62%
	Healthcare Providers	Year of publication	2019 or later	3	82	96.69 (89.97, 100.00)	8.04%
			2018 or prior	4	210	77.84 (37.64, 100.00)	95.20%
		Type of cancer*	Breast	4	70	91.18 (55.77, 100.00)	90.12%
			CRC	1	14	85.71 (61.59, 99.67)	NA
			Lung	1	57	96.49 (89.71, 99.94)	NA
			Prostate	1	151	50.33 (42.35, 58.31)	NA
		Sex	Females	4	70	91.18 (55.77, 100.00)	90.12%
			Both	3	222	79.98 (46.43, 99.49)	94.74%
Willingness to engage in personalised risk assessment	Participants (General Public)	Year of publication*	2019 or later	3[a]	2,870	94.82 (93.84, 95.72)	11.60%
			2018 or prior	3	6,876	70.02 (58.97, 80.00)	98.39%
		Type of cancer	Breast	5[a]	8,308	81.42 (66.12, 92.93)	99.47%
			Prostate	1	1,438	94.99 (93.80, 96.06)	NA
		Sex	Females	5[a]	8,308	81.42 (66.12, 92.93)	99.47%
			Males	1	1,438	94.99 (93.80, 96.06)	NA
		Region	Europe	5[a]	9,698	81.69 (66.37, 93.11)	99.62%
			North America	1	48	95.83 (87.85, 99.93)	NA
		Mean age of participants*	50 and less	3[a]	2,870	94.82 (93.84, 95.72)	11.60%
			Over 50	3	6,876	70.02 (58.97, 80.00)	98.39%
Satisfaction with PRBS	Participants (General Public)	Year of publication	2019 or later	1	48	79.17 (66.39, 89.64)	NA
			2018 or prior	2	194	94.52 (82.22, 100.00)	83.38%
		Region**	Europe	1	141	97.87 (94.68, 99.74)	NA
			North America	2	101	84.35 (74.01, 92.56)	39.16%
Intended adherence to recommended screening protocols	Participants (General Public)	Year of publication*	2019 or later	1	336	4.46 (2.49, 6.96)	NA
			2018 or prior	1	149	67.79 (60.04, 75.07)	NA
		Type of cancer*	Breast	1	149	67.79 (60.04, 75.07)	NA
			Prostate	1	336	4.46 (2.49, 6.96)	NA
		Sex*	Females	1	149	67.79 (60.04, 75.07)	NA
			Males	1	336	4.46 (2.49, 6.96)	NA
		Region*	Europe	1	149	67.79 (60.04, 75.07)	NA
			North America	1	336	4.46 (2.48, 6.96)	NA

		Mean age of participants*	50 and less	1	336	4.46 (2.48, 6.96)	NA
			Over 50	1	149	67.79 (60.04, 75.07)	NA
Intent to screen more	Participants (General Public)	Year of publication	2019 or later	3	3,764	94.52 (85.53, 99.35)	98.90%
			2018 or prior	7	10,646	81.09 (62.80, 94.24)	99.69%
		Type of cancer*	Breast	9	12,972	84.23 (70.09, 94.49)	99.68%
			Prostate	1	1,438	97.01 (96.06, 97.83)	NA
		Sex*	Females	9	12,972	84.23 (70.09, 94.49)	99.68%
			Males	1	1,438	97.01 (96.06, 97.83)	NA
		Region*	Asia-Pacific	1	205	84.88 (79.62, 89.48)	NA
			Europe	7	9,859	83.14 (64.44, 95.83)	99.73%
			North America	1	4,219	85.90 (84.83, 86.93)	NA
			West-Pacific	1	127	99.21 (96.55, 100.00)	NA
		Mean age of participants	50 and less	5	8,188	91.41 (84.04, 96.67)	98.88%
			Over 50	5	6,220	79.24 (51.71, 97.02	99.57%
Intent to screen less	Participants (General Public)	Year of publication	2019 or later	3	3,764	59.98 (38.31, 79.78)	99.05%
			2018 or prior	7	10,773	53.92 (43.34, 64.33)	98.86%
		Type of cancer*	Breast	9	13,099	53.30 (44.71, 61.80)	98.73%
			Prostate	1	1,438	83.03 (81.05, 84.93)	NA
		Sex*	Females	9	13,099	53.30 (44.71, 61.80)	98.73%
			Males	1	1,438	83.03 (81.05, 84.93)	NA
			Both	1	229	31.88 (25.99, 38.07)	NA
		Region**	Asia-Pacific	1	204	68.63 (62.08, 74.83)	NA
			Europe	7	9,758	53.81 (40.56, 66.78)	99.32%
			North America	1	4,219	49.30 (47.79, 50.81)	NA
			West-Pacific	1	127	71.65 (63.47, 79.19)	NA
		Mean age of participants	50 and less	5	8,187	65.00 (53.11, 76.02)	99.02%
			Over 50	5	6,350	47.69 (34.86, 60.67)	97.90%

Note: NA, not applicable.
[a]For Koitsalu 2016, the study population was split in Males and Females since data was provided for each cohort.
*p-value<0.0001.
**p-value =0.01.
